# MicroRNA-targeted reprogramming of CD8+ T cells against cancer

**DOI:** 10.3389/fimmu.2026.1770102

**Published:** 2026-04-24

**Authors:** Yuchen Mao, Yujin Liu, Kaiyan Jing, Yiqing Shao, Kangping Yang, Jiaqiang Wu, Xianhuan Zhou, Heng Wang, Ziling Fang

**Affiliations:** 1 Department of Oncology, The First Affiliated Hospital, Jiangxi Medical College, Nanchang University, Nanchang, Jiangxi, China; 2 The First Clinical Medical College, Jiangxi Medical College, Nanchang University, Nanchang, Jiangxi, China; 3 The Second Clinical Medical College, Jiangxi Medical College, Nanchang University, Nanchang, Jiangxi, China; 4 Department of Orthopedics, The First Affiliated Hospital, Jiangxi Medical College, Nanchang University, Nanchang, Jiangxi, China

**Keywords:** cancer, CD8+T cell, cellular pathway, immunology, microRNA

## Abstract

This scoping review highlights the critical role of microRNAs (miRNAs) in mediating the bidirectional crosstalk between CD8+ T cells and tumor cells within the immunosuppressive tumor microenvironment (TME). Specific miRNAs (e.g., miR-155, miR-340-5p) orchestrate CD8+ T cell function by fine-tuning immune checkpoints (PD-1/PD-L1), metabolic reprogramming, and epigenetic states. Conversely, CD8+ T cells influence tumor behavior via exosomal miRNA transfer (e.g., miR-765). Our analysis reveals both pan-cancer mechanisms, such as PD-1/PD-L1 regulation, and tissue-specific miRNA functions (e.g., miR-143 in melanoma). To overcome translational challenges like off-target effects, innovative delivery strategies using lipid nanoparticles and engineered exosomes are being developed. This review provides a mechanistic framework for miRNA-mediated interactions, offers clinical insights for novel combination therapies, and assesses future directions, thereby advancing the development of precision immunotherapies.

## 1 Introduction

Cancer remains the second leading cause of death globally. According to GLOBOCAN 2020 data, it caused approximately 9.96 million deaths worldwide in 2020, with lung cancer being the primary cause of cancer mortality (1). Recently, therapeutic strategies based on CD8+ T lymphocytes have highlighted the importance of immune responses in the fight against cancer.

CD8+ T cells are the core effector cells of the adaptive immune system. They recognize tumor-associated antigens bound to MHC class I complexes, release perforin and granzyme B to directly induce tumor cell apoptosis, and simultaneously secrete cytokines such as IFN-γ and TNF-α to enhance antitumor immune responses (2), (3).

Chimeric antigen receptor T-cell (CAR-T) therapy genetically engineers T cells to recognize specific tumor antigens, achieving unprecedented success in patients with relapsed/refractory B-cell acute lymphoblastic leukemia (R/R B-ALL), particularly among children and young adults. In the case of CD19-targeted CAR-T cells such as tisagenlecleucel, trials have demonstrated a complete remission (CR) rate of up to 81% in pediatric and young adult patients with R/R B-ALL (4).Adoptive cell therapy (ACT) involves the *in vitro* expansion and reinfusion of tumor-infiltrating lymphocytes (TILs). As a primary form of ACT, TIL therapy has demonstrated remarkable efficacy in metastatic melanoma, achieving an objective response rate consistently around 50% (5).However, these therapies have limited efficacy in solid tumors, primarily constrained by factors such as immune suppression in the TME, T-cell exhaustion, and tumor antigen heterogeneity.

Therapeutic strategies based on CD8+ T cells primarily include: Immune checkpoint blockers (ICBs) restore T-cell function by blocking PD-1/PD-L1 or CTLA-4 negative regulatory signals, fundamentally transforming the treatment landscape for advanced melanoma. For instance, monotherapy with anti-PD-1 agents (such as pembrolizumab or nivolumab) achieves objective response rates (ORR) of 33%–45% (6).While immune checkpoint inhibitors (ICIs) (7) have significantly improved outcomes for some patients, their objective response rates as monotherapy typically range only from 10% to 30%. However, concerns remain, as immune-related adverse events (irAEs) can emerge, including immune-mediated pneumonia, myocarditis, hepatitis, and colitis. Grade 3–4 irAEs occur in approximately 10%–15% of patients receiving anti-PD-1/PD-L1 monotherapy and can reach 30%–55% in regimens including ipilimumab. In addition to these safety concerns, acquired resistance is common even among initial responders, with median progression-free survival typically limited to 3–4 months (1).Therefore, elucidating the mechanisms of immune cell dysfunction within the TME is critical for developing novel therapeutic strategies.miRNA is a class of non-coding RNA approximately 18–25 nucleotides in length that suppresses gene expression at the post-transcriptional level or promotes mRNA degradation by binding to the 3’ untranslated region (3’UTR) of target mRNAs (8). Within the TME, miRNAs regulate CD8+ T cell function through multiple mechanisms. Tumor-derived miR-21 and miR-155-5p upregulate PD-1/PD-L1 expression, promoting T cell exhaustion (9). Conversely, miR-340-5p enhances T cell infiltration and cytotoxicity by targeting the immunosuppressive molecule CD73 (10). Simultaneously, activated CD8+ T cells can deliver specific miRNAs via exosomes to negatively regulate tumors: T cell-derived miR-765 targets tumor cell PLK1 to inhibit proliferation, while miR-298 suppresses tumor metastasis by downregulating the β-catenin signaling pathway (11), (12). This bidirectional miRNA-mediated regulatory network provides a crucial molecular basis for understanding tumor immune escape mechanisms and developing novel immunotherapy strategies.

Targeted molecular communication mediated by miRNAs between CD8+ T cells and tumor cells holds promise as a new strategy to overcome current bottlenecks in immunotherapy. However, despite the growing number of studies in this field, there is still a lack of comprehensive literature reviews to systematically map the knowledge landscape, core concepts, and potential research directions of this emerging research area. Given this, this review employs a scoping review methodology, conducting a comprehensive literature search on miRNA, CD8+ T cells, and cancer-related topics to comprehensively examine the current state of research on miRNA-mediated bidirectional regulatory networks between CD8+ T cells and tumor cells.

## 2 MicroRNA in regulating CD8+ T cells in cancer

### 2.1 Respiratory system

**Figure 1:**
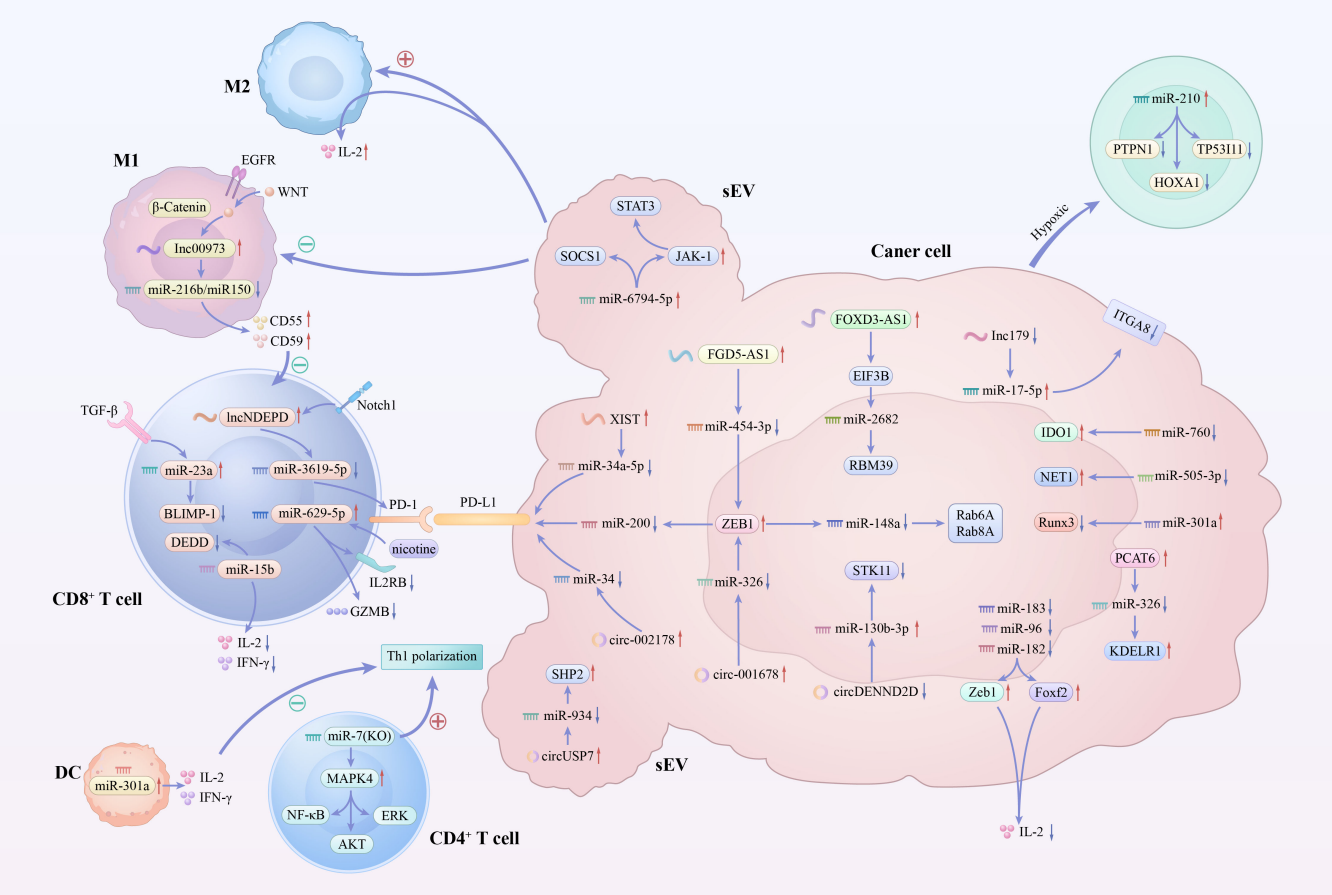
Schematic illustration of miRNA-mediated regulation of CD8^+^ T cell function in respiratory system tumors. In respiratory system tumors, miRNAs regulate CD8^+^T cell function through multiple key pathways, thereby promoting tumor immune escape and progression. In the PD-L1/PD-1 pathway, they alter PD-L1 expression to induce T cell exhaustion; in TGF-β signaling, they upregulate specific miRNAs to impair CD8^+^T cell differentiation and cytolytic activity. In metabolic/inflammatory pathways, miRNAs affect cytokine secretion and disrupt immune responses, while in the tumor microenvironment pathway, they regulate macrophage polarization and complement factors to block CD8^+^T cell activation. Collectively, these miRNA-mediated regulatory effects on CD8^+^T cells shape the immunosuppressive state of respiratory tumors.

The miRNA-mediated regulation of CD8+ T cell function in respiratory system tumors involves a complex network of multiple signaling pathways. As shown in **Figure 1**, in respiratory system tumors, miRNAs regulate CD8^+^T cell function through multiple key pathways, thereby promoting tumor immune escape and progression. In the PD-L1/PD-1 pathway, they alter PD-L1 expression to induce T cell exhaustion; in TGF-β signaling, they upregulate specific miRNAs to impair CD8^+^T cell differentiation and cytolytic activity. In metabolic/inflammatory pathways, miRNAs affect cytokine secretion and disrupt immune responses, while in the TME pathway, they regulate macrophage polarization and complement factors to block CD8^+^T cell activation. Collectively, these miRNA-mediated regulatory effects on CD8^+^T cells shape the immunosuppressive state of respiratory tumors. In lung malignancies and other respiratory system tumors, such as laryngeal cancer and nasopharyngeal cancer, miRNAs profoundly influence the number, activation status, and effector function of CD8+ T cells by regulating key immune checkpoint molecules, cytokines, and multiple signal transduction pathways, thereby participating in tumor immune escape and disease progression. **Table 1** presents the function of CD8+T cells by these miRNAs related to the respiratory system and the relevant information on subsequent tumor progression.

**Table 1:** miRNA-mediated regulation of CD8^+^ T cell function in respiratory system tumors.

MicroRNA	Source	Express	Pathway	Cancer	CD8+T cell	Result	Ref.
miR-34a-5p	cancer cell	↓	XIST↑/miR-34a-5p↓/PD-L1↑	LUNG	↓	viability, apoptosis, migration, and invasion of cancer cells↑	(13)
miR-34	cancer cell	↓	circ002178↑/miR-34↓/PD-L1↑	LUAD	↓	T-cell exhaustion↑	(14)
miR-200	cancer cell	↓	ZEB1↑/miR-200↓/PD-L1↑	NSCLC	↓	resistance to treatment with PD-L1 antagonists ↑	(15)
miR-326	cancer cell	↓	circ001678↑/miR-326↓/ZEB1↑/PD-L1↑	NSCLC	↓	immune escape and malignant progression of NSCLC↑	(16)
miR-454-3p	cancer cell	↓	FGD5-AS1↑/miR-454-3p↓/ZEB1↑/PD-L1↑	NSCLC	↓	proliferation, invasion and angiogenesis of cancer cells↑	(17)
miR-2682	exosome	↓	FOXD3-AS1↑/EIF3B↓/miR-2682↓/RBM39↑	LUNG	↓	cancer cell growth and inhibition of apoptosis↑	(18)
miR-3619-5p	CD8^+^ T cell	↓	Notch1/lncNDEPD1↑/miR-3619-5p↓/PD-L1↑	NSCLC	↓	tumoricidal effects of chimeric Ag receptor T cells↓	(19)
miR-23a	CD8^+^ T cell	↑	TGF-β↑/miR-23a↑/BLIMP-1↓	LUNG	↓	tumor immunotherapy↓ and tumor immune-evasion↑	(20)
miR-15b	CD8^+^ T cell	↑	DEDD↓ IL-2, IFN-γ, CD69↓ and CD44↑	LUNG	↓	anti-tumor immunity through inhibiting function of CD8+ T cells↓	(21)
miR-301a	DC	↑	IL-12↓/IFN-γ↓	LUNG	↓	altered antigen-specific T-helper cytokine profile	(22)
miR-629-5p	CD8^+^ T cell	↑	nicotine/miR-629-5p↑/IL2RB, GZMB↓	NSCLC	↓	granzyme B level↓	(23)
miR-210	Hypoxic cancer cell	↑	PTPN1, HOXA1, TP53I11↓	NSCLC/SKCM	↓	resistance in hypoxic tumor targets to CD8+ T cell -mediated lysiss↑	(24)
miR-301a	cancer cell	↑	Runx3↓	LUNG	↓	TME antitumor immunity↓	(25)
miR-216b/150	M1 macrophages	↓	EGFR/Wnt/β-catenin/lnc00973↑/miR-216b,150↓/CD55, CD59↑	LUAD	↓	complement system and cytokine secretion required for CD8+ T cell activation↓	(26)
miR-6794-5p	exosome	↑	SOCS1↓ JAK1-STAT3↑	GBM/LUAD	↓	M1 macrophages↓ and M2 macrophages↑ IL-10 secreted from M2 macrophages↑	(27)
miR-130b-3p	cancer cell	↑	circDENND2D↓/miR-130b-3p↑/STK11↓	NSCLC	↓	proliferation, migration, invasion and immune escape of cancer cell↑	(28)
miR-30a	iTreg	↓	SOCS1↓/Akt-STAT1	LUNG	↑	inhibitory effects of iTregs on CD4+ and CD8+ T cells↓	(29)
miR-760	cancer cell	↓	IDO1↑	NSCLC	↓	immune tolerance↑	(30)
miR-24-3p	exosome	↑	FGF11↓/P-ERK, P-STAT1, P-STAT3↑ and P-STAT5↓	NPC	↓	T-cell proliferation and Th1 and Th17 differentiation↓ and induction of Tregs↑	(31)
miR-7	CD4^+^ T cell	–	miR-7(KO)/MAPK4↑/NF-κB, AKT, ERK	LUNG	↑	infiltration, proliferation, activation and Th1 polarization of CD4+ T cells↑	(32)
miR-193b	cancer cell	↑	IL-10(+)MOS↑	LC	↓	CD8+ T cell activities↓	(33)
miR-21	cancer cell	↑	TLR3↑/miR-21↑/NFI-A↑/IL-10(+)B cell↑	NPC	↓	CD8+ T cell activities↓	(34)
miR-934	exosome	↓	circUSP7↑/miR-934↓/SHP2↑	NSCLC	↓	resistance to anti-PD1 immunotherapy↑	(35)
miR-148a	cancer cell	↓	ZEB1↑/miR-148a↓/Rab6A, Rab8A↑	LUAD	↓	antitumor immunity and resistance to PD-L1 ICB↓	(36)
miR-505-3p	cancer cell	↓	NET1↑	LUAD	↓	expression of immune checkpoint molecules in tumor cells↑	(37)
hsa-miR-19b-5p/19b-2-5p/379-5p	cancer cell/CD4^+^ and CD8^+^ T cell	↓	PCED1B-AS1↑/hsa-miR-19b-5p, hsa-miR-19b-2-5p↓/GPR174↑ RP11-81H14.2↑/hsa-miR-379-5p↓/CD226↑	LUAD	↓	migration, invasion and metastasis of tumor cells↑	(38)
hsa-miR-126/218/30a/145/1/195/551b/497/101 hsa-miR-215/31/196a/21	cancer cell	↓/↑	hsa-miR-218/COL1A1↑ hsa-miR-145/PECAM1↓ hsa-miR-1/EDN1↓ hsa-miR-195/VWF↓, COL1A1↑, COL3A1↑ hsa-miR-497/VWF↓, COL1A1↑ hsa-miR-101/CDH5↓ hsa-miR-215/TEK↓ hsa-miR-31/COL1A1↑, VWF↓, TEK↓ hsa-miR-196/COL1A1↑, COL3A1↑	LUAD	↓	low PECAM1 expression was an important independent predictor of unfavorable LUAD prognosis	(39)
miR-183/96/182	cancer cell	↓	ZEB1, Foxf2↑/IL-2↓	LUAD	↓	cancer progression and metastasis↑	(40)
miR-17-5p	cancer cell	↑	lnc0179↓/miR-17-5p↑/ITGA8↓	LUAD	↓	cancer stemness↑	(41)
hsa-miR-326	cancer cell	↓	PCAT6↑/hsa-miR-326↓/KDELR1↑	LUAD	↓	poor prognosis of cancer↑	(42)

This table summarizes the regulatory roles of various microRNAs (miRNAs) in the tumor microenvironment, primarily focusing on lung cancer. It details how miRNAs from different cellular sources (e.g., cancer cells, CD8+ T cells, exosomes) modulate key pathways—notably the PD-1/PD-L1 axis—to influence CD8+ T cell function and promote tumor immune evasion, progression, and resistance to immunotherapy. Arrows (↑/↓) indicate upregulation or downregulation of expression or activity.

LUNG, lung cancer; LUAD, lung adenocarcinoma; NSCLC, non-small cell lung cancer; SKCM, melanoma; GBM, glioblastoma; NPC, nasopharyngeal carcinoma; LC laryngeal cancer. Expression column: ↑, overexpression (upregulation); ↓, downregulation (suppression of expression). CD8^+^ T cell column: ↑, change that confers a benefit to CD8^+^ T cells (e.g., enhanced function, survival, or proliferation); ↓, change that is harmful to CD8^+^ T cells (e.g., impaired function or reduced viability).

#### 2.1.1 The PD-L1/PD-1 immune checkpoint pathway

This pathway directly suppresses the antitumor function of CD8^+^ T cells by upregulating the expression of the immune checkpoint molecule PD-L1. The core mechanism involves activating the transcription factor ZEB1 and regulating non-coding RNAs, such as circular RNAs (circRNAs) and long non-coding RNAs (lncRNAs), to form a cascade inhibition network. For instance, the downregulation of miRNA 34a-5p increases PD-L1 expression and promotes T cell exhaustion (13, 14), while miRNAs such as 200, 454-3p, and 326 enhance tumor immune escape through the ZEB1-PD-L1 axis (15–17). Additionally, miR-2682 is downregulated in exosomes, which leads to the upregulation of FOXD3-AS1 and EIF3B, ultimately increasing PD-L1 expression and inhibiting cancer cell apoptosis (18). Furthermore, downregulation of miR-3619-5p that expressed by CD8^+^ T cells, induces PD-L1 overexpression, weakening CAR-T therapy efficacy (19). This indicates that the pathway plays a pivotal role in cell-intrinsic and microenvironmental regulation.

#### 2.1.2 The TGF-β signaling pathway

TGF-β signaling sculpts an immunosuppressive microenvironment by regulating miRNA expression. In CD8^+^ T cells, miR-23a is upregulated by TGF-β, thereby inhibiting the transcription factor BLIMP-1 (20). BLIMP-1 is a key regulator of T cell effector function, and its downregulation leads to impaired CD8^+^ T cell differentiation and reduced cytolytic activity, ultimately diminishing the response to tumor immunotherapy. This pathway highlights the direct regulatory role of the TGF-β-miRNA axis in T cell dysfunction.

#### 2.1.3 The metabolic/inflammatory factor regulatory pathway

This pathway targets core effector molecules of the immune response (such as IL-2, IFN-γ, and granzyme B) by mediating their expression imbalance via miRNAs. miR-15b derived from CD8^+^ T cells inhibits DEDD, reduces IL-2/IFN-γ secretion, and weakens the cell activation marker CD69 (21); while miR-301a in DC cells inhibits IL-12/IFN-γ, disrupting Th1-type immune responses (22). Notably, environmental factors (such as nicotine) can induce the upregulation of miR-629-5p, directly inhibiting the expression of IL2RB and granzyme B (23), revealing the mechanism by which exogenous irritation influences T cell function through miRNAs. Additionally, miR-210 is upregulated in hypoxic cancer cells, targeting PTPN1, HOXA1, and TP53I11, which enhances resistance to hypoxic tumor targets to CD8+ T cell-mediated lysis (24).

#### 2.1.4 The tumor microenvironment remodeling pathway

This pathway regulates macrophage polarization and the complement system to remodel the immunosuppressive microenvironment. Cancer cell-derived miR-301a suppresses Runx3, thereby weakening the anti-tumor immune response in the microenvironment (25); meanwhile, the downregulation of miR-216b/150 in M1 macrophages causes the overexpression of complement inhibitory factors CD55/CD59, which blocks the complement signals required for CD8^+^ T cell activation (26). Exosomal miR-6794-5p promotes M2 macrophage polarization and IL-10 secretion through the SOCS1-JAK1-STAT3 axis (27), formulating a multifaceted immunosuppressive network. Moreover, miR-130b-3p is upregulated in cancer cells, targeting STK11 and promoting cancer cell proliferation, migration, and invasion, as well as immune escape (28).

#### 2.1.5 The immune tolerance/Treg pathway

This pathway mediates immune tolerance through the activation of regulatory T cell (Treg) function or the regulation of tryptophan metabolism. Downregulation of miR-30a in iTreg cells relieves inhibition of SOCS1, enhancing Treg inhibitory function through the Akt-STAT1 signaling pathway (29). Tumor cells activate IDO1 by downregulating miR-760, promoting the accumulation of tryptophan metabolites and inducing T cell tolerance (30). Exosome miR-24-3p further promotes Treg differentiation and inhibits effector T cell proliferation by inhibiting FGF11 and imbalanced STAT signaling (31), thereby establishing systemic immune tolerance.

#### 2.1.6 The other key pathways

miR-7 promotes CD4^+^ T cell polarization toward Th1 via the MAPK4-NF-κB/AKT/ERK axis, indirectly enhancing CD8^+^ T cell activity (32); miR-193b/miR-21 inhibit CD8^+^ T cell function by inducing IL-10^+^ monocytes (33) or IL-10^+^ B cells (34), respectively; miR-934 induces PD-1 inhibitor resistance via the circUSP7-SHP2 axis (35), suggesting the role of non-canonical pathways in immune therapy resistance.

Additionally, miR-148a is downregulated in lung adenocarcinoma (LUAD), which leads to the upregulation of ZEB1 and Rab6A, enhancing tumor immune escape through the PD-L1 axis (36). miR-326 is downregulated in LUAD, which increases the expression of circ001678 and ZEB1, promoting immune escape and malignant progression (16). miR-505-3p is downregulated in LUAD, leading to the upregulation of NET1 and the downregulation of immune checkpoint molecules in tumor cells (37). hsa-miR-19b-5p/19b-2-5p/379-5p is downregulated in LUAD, which increases the expression of GPR174 and CD226, promoting the migration, invasion, and metastasis of tumor cells (38). hsa-miR-126/218/30a/145/1/195/551b/497/101 is downregulated in LUAD, which increases the expression of COL1A1 and COL3A1, promoting tumor progression and metastasis (39). miR-183/96/182 which increases the expression of ZEB1 and Foxf2, has the similar result (40). miR-17-5p is upregulated in LUAD, which increases the expression of lnc0179 and ITGA8, promoting cancer stemness (41). hsa-miR-326 is downregulated in LUAD, which increases the expression of KDELR1, promoting poor prognosis of cancer (42).

### 2.2 Digestive system

**Figure 2:**
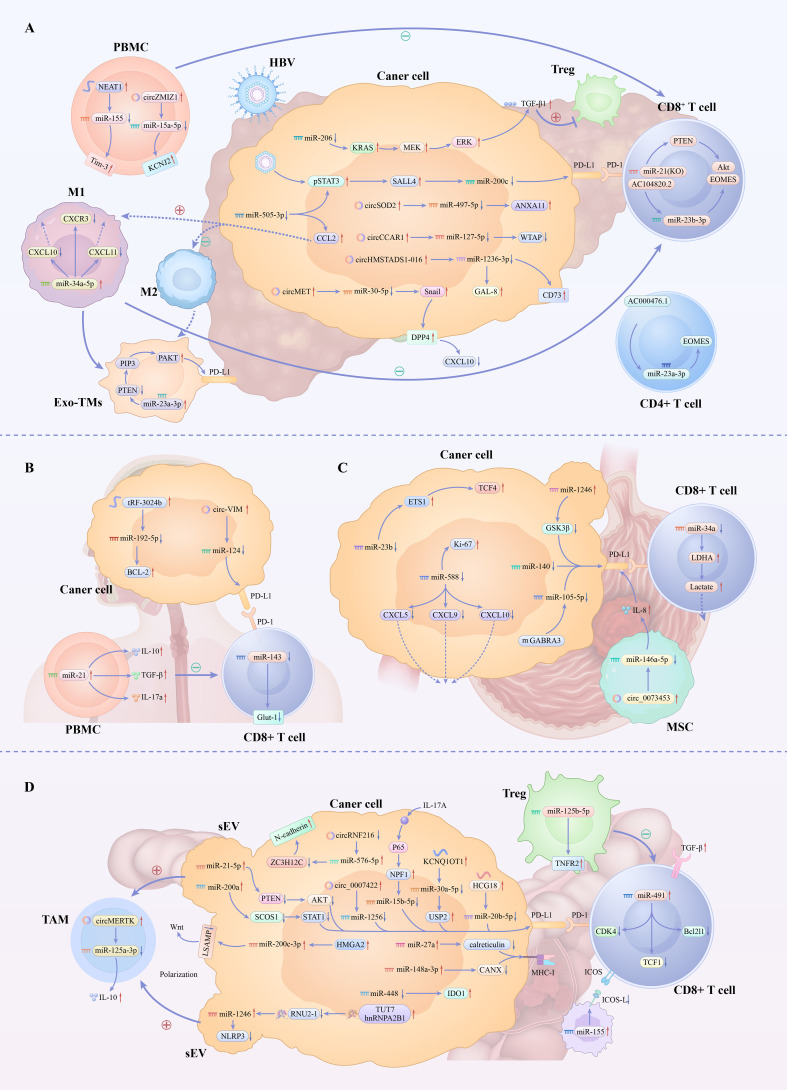
Schematic representation of miRNA-mediated regulation of the immune microenvironment in digestive system tumors. In liver cancer **(A)**, interactions among HBV, PBMCs, macrophages, and T cells involve miRNAs like miR-200c and miR-34a-5p, regulating PD-L1, T cell function, and macrophage polarization. Gastric cancer **(B)** sees miRNAs such as miR-21 and miR-143 modulate PD-L1 expression and CD8^+^ T cell differentiation. Esophageal cancer **(C)** utilizes miRNAs like miR-34a and miR-146a-5p in PD-L1 signaling and T cell metabolic reprogramming. Pancreatic cancer **(D)** relies on sEV-delivered miRNAs to regulate TAM polarization, Treg function, and PD-L1 expression in tumor cells, collectively forming immunosuppressive microenvironments through cellular crosstalk and shared mechanisms like PD-L1 upregulation and T cell exhaustion.

In recent years, miRNAs have been discovered to play a key regulatory role in the remodeling of the immune microenvironment in digestive system tumors. By precisely regulating multiple immune critical nodes such as immune checkpoint molecules, antigen presentation, T cell function, and macrophage polarization, they construct an immunosuppressive microenvironment conducive to tumor growth. Different digestive system tumors exhibit unique miRNA expression profiles and regulatory patterns, which also share common immune escape mechanisms, such as PD-L1 upregulation, T cell exhaustion, and the accumulation of immune-suppressive cells. **Figure 2** demonstrates miRNA-mediated immune microenvironment remodeling in diverse digestive system tumors. As shown in **Table 2**, miRNAs play different roles in the regulation of the immune microenvironment of digestive system tumors. These complex regulatory networks can be categorized into several primary mechanisms:

**Table 2:** miRNA-mediated regulation of CD8^+^ T cell function in digestive system tumors.

MicroRNA	Source	Express	Pathway	Cancer	CD8+T cell	Result	Ref.
miR-27a	cancer cell	↑	calreticulin↓/MHC-I↓	COADREAD	↓	distant metastasis and poor prognosis↑	(84)
miR-148a-3p	cancer cell	↑	CANX↓/MHC-I↓	COADREAD	↓	CD8+ T-cell-mediated immune attack *in vitro* and *in vivo*↓	(85)
miR-576-5p	cancer cell	↑	circRNF216↓/miR-576-5p↑/ZC3H12C↓/N-cadherin↑	COADREAD	↓	infiltration level of CD8+ T cells↓	(87)
miR-21-5p/200a	sEVs	↑	PTEN↓/AKT↓/PD-L1↑ SCOS1↓/STAT1↓/PD-L1↑	COADREAD	↓	macrophage M2 like polarization↑	(43)
miR-1246	exosome	↑	TUT7, hnRNPA2B1↑/RNU2-1↓/miR-1246↑/NLRP3↓	COADREAD	↓	polarization of TAMs↑	(59)
miR-125a-3p	TAM	↓	circMERTK↑/miR-125a-3p↓/IL-10↑	COADREAD	↓	the immunosuppressive activity of TAM-like cell↑	(60)
miR-15b-5p	cancer cell	↓	IL-17A/P65/NRF1↑/miR-15b-5p↓/PD-L1↑	COADREAD	↓	resistance to anti-PD-1 therapy↑	(44)
miR-30a-5p	–	↓	KCNQ1OT1↑/miR-30a-5p↓/USP2↑/PD-L1↑	COADREAD	↓	immune escape of cancer cells↑	(45)
miR-20b-5p	cancer cell	↓	HCG18↑/miR-20b-5p↓/PD-L1↑	COADREAD	↓	progress of the tumor and conferred to cetuximab resistance↑	(46)
miR-1256	cancer cell	↓	circ0007422↑/miR-1256↓/PD-L1↑	COADREAD	↓	proliferation, invasion, self-replication ability, and immune escape↑ and apoptosis of CRC cells↓	(47)
miR-491	CD8+ T cell	↑	TGF-β↑/miR-491↑/CDK4, TCF1, Bcl2l1↓	COADREAD	↓	production of interferon-γ in CD8+ T cells↓	(68)
miR-448	cancer cell	↓	IDO1↑	COAD	↓	immune tolerance↑	(74)
miR-125b-5p	Tregs	↓	TNFR2↑	COAD	↓	immunosuppressive activity of Tregs↑	(75)
miR-200c-3p	cancer cell	↑	HMGA2↑/miR-200c-3p↑/LSAMP↓/Wnt↑	COAD	↓	immunotherapy and overall survival↓	(77)
miR-155	B cell	↑	IcosL↓	COAD	↓	infiltration of tumors by cytotoxic T cells↓	(76)
miR-192-5p	cancer cell	↓	tRF-3024b↑/miR-192-5p↓/BCL-2↑	ESCC	↓	drug resistance against ICIs↑	(57)
miR-143	Tcm CD8+ cell	↓	Glut-1↓	ESCA	↓	differentiation of Tcm CD8+ cells and secretion of pro-inflammatory cytokines↑	(64)
miR-124	cancer cell	↓	circVIM↑/miR-124↓/PD-L1↑	ESCA	↓	immune escape and multiple malignant phenotypes of cancer cells↑	(56)
miR-21	PBMC	↑	IL‐10, TGF‐β, IL‐17a↑	ESCA	↓	population of CD8+ Tregs↑	(78)
miR-34a	CD8+ T cell	↓	LDHA↑/lactate↑	STAD	↓	number of Th1 cells and CD8+ T cell↓	(65)
miR-588	cancer cell	↓	Ki-67↑ CXCL5, CXCL9, CXCL10↓	STAD	↓	tumor cell proliferation↑ and overall survival↓	(67)
miR-105-5p	cancer cell	↓	GABRA3(methylation)/miR-105-5p↓/PD-L1↑	STAD	↓	immunogenicity of cancer cells↓	(48)
miR-146a-5p	MSC	↓	circ0073453↑/miR-146a-5p↓/IL-8↑/PD-L1↑	STAD	↓	migration and invasion of cancer cells↑	(49)
miR-1246	sEVs	↑	GSK3β↓/PD-L1↑	STAD	↓	lymphangiogenesis and lymph node immunosuppression↑	(50)
miR-140	cancer cell	↓	PD-L1↑	STAD	↓	myeloid−derived suppressive and Treg infiltration↑	(51)
miR-23b	cancer cell	↓	ETS1↑/TCF4↑	STAD	↓	the cell proportion of B memory cell, CD8+ T cell and CD4 naïve↓	(66)
miR-200c	cancer cell	↓	HBV/pSTAT3↑/SALL4↑/miR-200c↓/PD-L1↑	LIHC	↓	virus-induced immune exhaustion↑	(52)
miR-155	PBMC	↓	NEAT1↑/miR-155↓/Tim-3↑	LIHC	↓	the cytolysis activity against HCC↓	(69)
miR-21	CD8+ T cell	–	miR-21(KO)/PTEN/Akt	LIHC/fibrosarcoma	↓	proliferation and cytokine production of CD4+, CD8+ T cells↓	(70)
miR-30-5p	cancer cell	↓	circMET↑/miR-30-5p↓/Snail↑/DPP4↑/CXCL10↓	LIHC	↓	immune tolerance↑	(79)
miR-497-5p	cancer cell	↓	circSOD2↑/miR-497-5p↓/ANXA11↑	LIHC	↓	immune evasion and anti-PD-1 resistance↑	(80)
miR-127-5p	cancer cell	↓	circCCAR1↑/miR-127-5p↓/WTAP↑	LIHC	↓	immunosuppression and anti-PD1 resistance↑	(81)
miR-23a-3p	Exo-TMs	↑	PTEN↓/phosphatidylinositol 3/pAKT↑/PD-L1↑	LIHC	↓	PD-L1 expression in macrophages↑	(53)
miR-34a-5p	M1 macrophage	↑	CXCL10, CXCL11, CXCR3↓	LIHC	↓	Th1 cell polarization, activation of CD8+T, NK and NKT cells↓	(61)
miR-206	KC	↑	KLF4/CCL2/CCR2	LIHC	↑	M1 polarisation of macrophages and recruit CD8+ T cell↑	(63)
miR-206	cancer cell	↓	KRAS-MEK-ERK↑/TGFβ1↑	LIHC	↓	suppressor function and expansion of Tregs↓	(83)
miR-93-5p	LPC	↑	APC/GAL-9↑/AEBP2/H3K4me3/H3K27me3	LIHC	↓	immune escape and tumorigenesis↑	(82)
has-miR-23b-3p	activated memory CD4+ T cell/CD8+ T cells	–	AC104820.2-has-miR-23b-3p-EOMES AC000476.1-has-miR-23a-3p-EOMES	LIHC	–	up-regulated expression of T-cell related genes including EOMES, CST7 and CD5L indicates a favorable prognosis	(72)
miR-505-3p	cancer cell	↓	MELK↑/pSTAT3, CCL2↑	LIHC	↓	M1 macrophage polarization↑, M2 macrophage polarization↓	(62)
miR-15a-5p	PBMC	↓	circZMIZ1↑/miR-15a-5p↓/KCNJ2↑	LIHC	↓	apoptosis↑ and cytotoxicity↓ of CD8+ T cells	(71)
miR-1236-3p	cancer cell	↓	circHMSTADS1-016↑/miR-1236-3p↓/CD73, GAL-8↑	ICC	↓	effect of anti-PD1 treatment↓	(58)
miR-195-5p	cancer cell/CD8+ T cell	↓	lnc00473↑/miR-195-5p↓/PD-L1↑	PAAD	↓	apoptosis, proliferation, invasion, and migration of the cancer cells↓	(54)
miR-429	cancer cell	↑	–	PAAD	↑	CD4+ and CD8+ T cell infiltration↑ numbers of Tregs↓	(73)
miR-194-5p	cancer cell	↓	PD-L1↑/IFN-γ↓	PDAC	↓	survival rate of patients↓	(55)

This table summarizes the role of various microRNAs (miRNAs) in regulating CD8^+^ T cell function across digestive system cancer types. It details miRNA sources, expression changes (↑/↓), key molecular pathways, and their impacts on CD8^+^ T cell activity and anti-tumor immunity. The data highlight mechanisms such as PD-L1 upregulation and T cell exhaustion that contribute to immune evasion and immunotherapy resistance.

COADREAD, colorectal adenocarcinoma; COAD, colon adenocarcinoma; ESCC, esophageal squamous cell carcinoma; ESCA, esophageal carcinoma; STAD, stomach adenocarcinoma; LIHC, liver hepatocellular carcinoma; ICC, intrahepatic cholangiocarcinoma; PAAD, pancreatic adenocarcinoma; PDAC, pancreatic ductal adenocarcinoma. Expression column: ↑, overexpression (upregulation); ↓, downregulation (suppression of expression). CD8^+^ T cell column: ↑, change that confers a benefit to CD8^+^ T cells (e.g., enhanced function, survival, or proliferation); ↓, change that is harmful to CD8^+^ T cells (e.g., impaired function or reduced viability).

#### 2.2.1 The PD-L1/PD-1 immune checkpoint pathway

The upregulation of the PD-L1/PD-1 axis is a prevalent miRNA-driven mechanism across digestive cancers, leading to profound T cell exhaustion. In colorectal cancer, exosome-derived miR-21-5p/200a dual-upregulates PD-L1 through the PTEN/AKT and SCOS1/STAT1 pathways (43). Similarly, miR-15b-5p (44), miR-30a-5p (45), miR-20b-5p (46), and miR-1256 (47) are downregulated by lncRNAs, leading to the upregulation of PD-L1 and mediating treatment resistance. Gastric cancers exhibit multiple pathways for immune checkpoint activation: low expression of miR-105-5p upregulates PD-L1 through GABRA3 methylation (48); MSC-derived miR-146a-5p downregulation promotes tumor migration through the circ0073453/IL-8/PD-L1 axis (49); sEVs-delivered miR-1246 drives lymph node immunosuppression through GSK3β/PD-L1 (50); and low expression of miR-140 in HP-associated gastric cancer directly upregulates PD-L1 (51). In the liver, HBV inhibits miR-200c through the pSTAT3/SALL4 axis and upregulates PD-L1 to induce immune exhaustion (52), while Exo-TMs-derived miR-23a-3p upregulates macrophage PD-L1 (53, 54). Pancreatic and esophageal cancers also heavily rely on this axis; for example, miR-195-5p (54) and miR-194-5p (55) mediate immune suppression via PD-L1 pathways, and in esophageal cancer, low expression of miR-124 promotes immune escape through the circVIM/PD-L1 axis (56). Furthermore, downregulation of miR-192-5p in ESCC (57) and low expression of miR-1236-3p in intrahepatic cholangiocarcinoma (58) mediate immune checkpoint inhibitor resistance.

#### 2.2.2 Tumor microenvironment and macrophage polarization

miRNAs actively reshape the TME by polarizing macrophages toward an immunosuppressive phenotype and recruiting regulatory cells. In colorectal cancer, exosome-derived miR-21-5p/200a drives macrophage M2 polarization (43), while miR-1246 inhibits NLRP3 to promote tumor-associated macrophage (TAM) polarization (59). Meanwhile, the downregulation of miR-125a-3p in TAMs enhances immunosuppression via the circMERTK/IL-10 axis (60). The liver microenvironment relies heavily on macrophage modulation: high expression of miR-34a-5p in M1 macrophages inhibits CXCL10/11/CXCR3, thereby weakening CD8^+^/NK cell activity (61); conversely, low expression of miR-505-3p promotes M1 polarization (62). Additionally, miR-206 in Kupffer cells recruits CD8^+^ T cells through KLF4/CCL2/CCR2 (63). In gastric cancer, low expression of miR-140 increases the infiltration of myeloid-derived suppressor cells (MDSCs) (51).

#### 2.2.3 Direct regulation of T cell function, metabolism, and differentiation

Numerous miRNAs target intrinsic pathways within CD8^+^ T cells, compromising their functional and metabolic states. In esophageal cancer, the downregulation of miR-143 in central memory CD8^+^ T cells inhibits Glut-1 expression, critically impairing T cell differentiation and pro-inflammatory factor secretion (64). Gastric cancer exhibits similar suppressive tactics: low expression of miR-34a in CD8^+^ T cells reduces Th1 and CD8^+^ populations via the LDHA/lactic acid axis (65), and miR-23b downregulation reduces the proportion of CD8^+^ and CD4^+^ naive T cells through ETS1/TCF4 (66). Furthermore, the downregulation of miR-588 leads to the upregulation of Ki-67 and downregulation of CXCL5/9/10, promoting proliferation and reducing survival rates (67). In colorectal cancer, high expression of miR-491 in CD8^+^ T cells is induced by TGF-β, inhibiting CDK4/TCF1/Bcl2l1 and reducing IFNγ secretion (68). Liver cancers suppress T cells through mechanisms such as low miR-155 reducing cytotoxicity via the NEAT1/Tim-3 axis (69), miR-21 deficiency inhibiting proliferation through PTEN/Akt (70), and the downregulation of miR-15a-5p increasing CD8^+^ T cell apoptosis (71). Conversely, some miRNAs indicate a favorable prognosis or anti-tumor effect, such as upregulated hsa-miR-23b-3p/23a-3p in memory T cells enhancing EOMES expression (72), and high expression of miR-429 in pancreatic cancer increasing CD4^+^/CD8^+^ T cell infiltration (73).

#### 2.2.4 Immune tolerance and regulatory T cell modulation

miRNAs foster immune tolerance by activating Treg functions and modulating metabolic pathways like tryptophan degradation. In colorectal cancer, specific regulation is closely associated with Treg function: low expression of miR-448 leads to the upregulation of IDO1 (74), downregulation of miR-125b-5p in Tregs enhances immunosuppressive activity through TNFR2 (75), and elevated B cell-derived miR-155 inhibits IcosL to reduce cytotoxic T cell infiltration (76). High expression of miR-200c-3p also reduces the effect of immune therapy through the HMGA2/LSAMP/Wnt axis (77). Esophageal cancers demonstrate high expression of miR-21 in PBMCs, which induces IL-10/TGF-β/IL-17a secretion, increasing the proportion of CD8^+^ Tregs (78). In gastric cancer, low miR-140 increases Treg infiltration (51). Liver cancers utilize miR-30-5p (79), miR-497-5p (80), and miR-127-5p (81) to promote immune tolerance through various circRNA/miRNA axes, and high expression of miR-93-5p promotes immune escape through the epigenetic regulation of APC/GAL-9/AEBP2 (82). However, low expression of miR-206 in liver cancer cells can inhibit Treg function through KRAS-MEK-ERK/TGFβ1 (83).

#### 2.2.5 Antigen presentation deficiencies and infiltration barriers

The initial recognition of tumors by CD8^+^ T cells is also heavily regulated. In colorectal cancer, elevated expression of miR-27a downregulates calretin and MHC-I molecules, promoting distant metastasis and poor prognosis (84). Similarly, miR-148a-3p reduces MHC-I expression by inhibiting CANX, thereby weakening the *in vivo* and *in vitro* antitumor activity of CD8^+^ T cells (85, 86). Beyond antigen presentation, miRNAs can establish physical or chemical barriers; for instance, miR-576-5p upregulates N-cadherin via the circRNF216/ZC3H12C axis, actively reducing CD8^+^ T cell infiltration into the tumor site (87).

### 2.3 Gynecology

**Figure 3:**
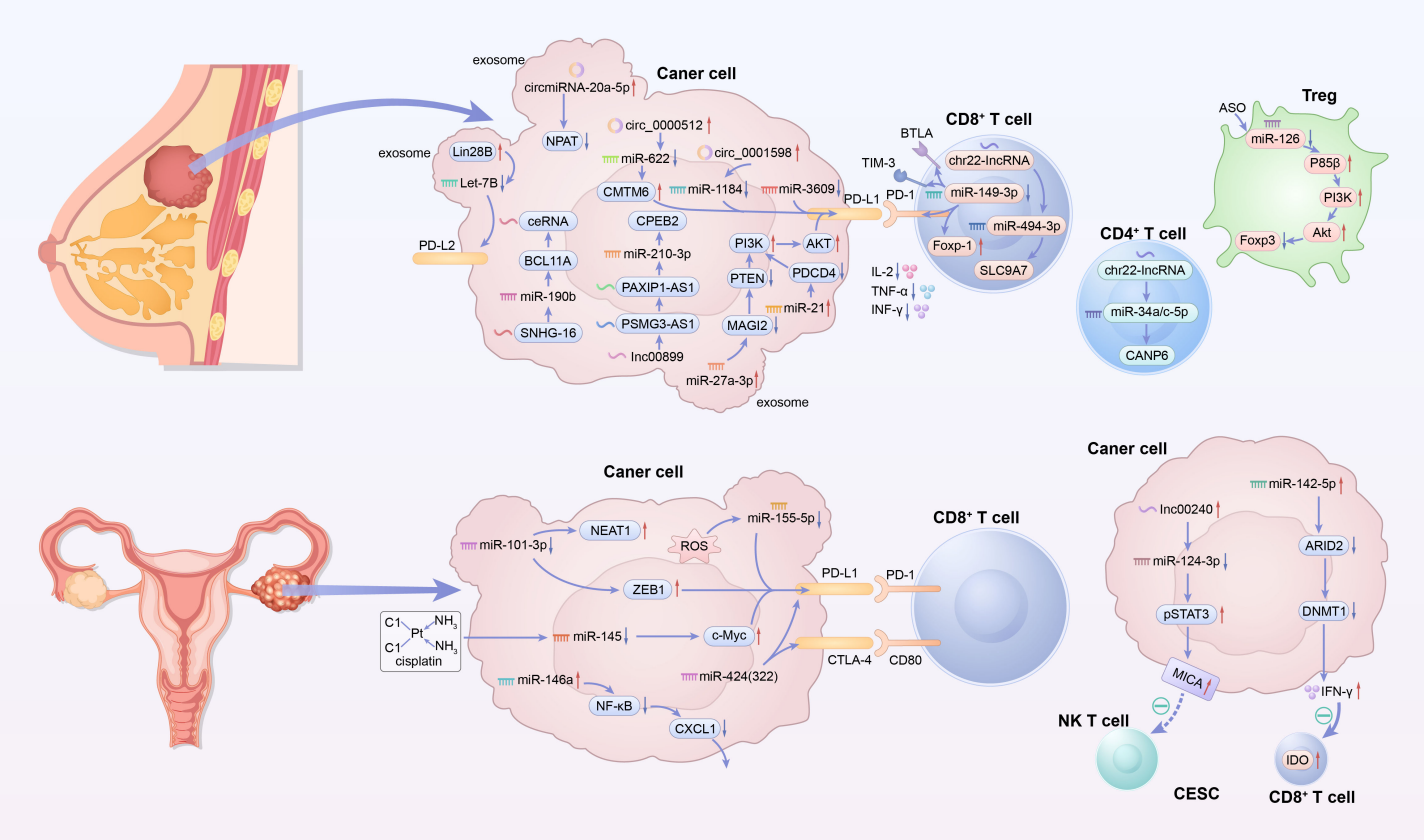
Schematic illustration of miRNA-mediated immune regulation in gynecological tumors. The upper section focuses on breast cancer, with tumor cells releasing exosomes containing circRNAs/miRNAs (e.g., cirmiR-20a-5p, miR-149-3p) to interact with CD8^+^ T cells, CD4^+^ T cells, and Tregs, involving pathways like PD-L1/PD-1. The lower section depicts other gynecological tumors (e.g., ovarian, cervical), where tumor cells use miRNAs (e.g., miR-101-3p, miR-142-5p) to regulate PD-L1/CTLA-4 and impair CD8^+^ T cell, NK T cell functions, driving immune escape. Expression column: ↑, overexpression (upregulation); ↓, downregulation (suppression of expression). CD8^+^ T cell column: ↑, change that confers a benefit to CD8^+^ T cells (e.g., enhanced function, survival, or proliferation); ↓, change that is harmful to CD8^+^ T cells (e.g., impaired function or reduced viability).

The miRNA-mediated immune regulatory mechanisms in gynecological tumors exhibit high complexity and heterogeneity, forming a key regulatory network for tumor immune escape. Although these regulatory mechanisms vary across different tumor types, they all focus on core components: T cell function regulation, activation of the PD-L1/PD-L2 immune checkpoint pathway, and the formation of treatment resistance. **Figure 3** illustrates miRNA-mediated immune regulation in gynecological tumors. **Table 3** summarized the highly heterogeneous complexity and heterogeneity of miRNAs associated with gynecological tumors according to the classification of different gynecological tumors.

**Table 3:** miRNA-mediated regulation of CD8^+^ T cell function in gynecology tumors.

MicroRNA	Source	Express	Pathway	Cancer	CD8+T cell	Result	Ref.
miR-126	Treg	↑	ASO/miR-126↓/p85β↑/PI3K-Akt↑/Foxp3↓	BRCA	↑	induction and suppressive function of Tregs↓	(106)
miR-149-3p	CD8+ T cell	↓	PD-1, TIM-3, BTLA, Foxp1↑	BRCA	↓	secretion of effector cytokines (IL-2, TNF-α, IFN-γ) for T cell activation↓	(99)
miR-34a miR-494-3p	CD4+ T cell CD8+ T cell	↓/↑	chr22-38_28785274-29006793.1-miR-34a/c-5p-CAPN6 chr22-38_28785274-29006793.1-miR-494-3p-SLC9A7	BRCA	↓	cellular activities associated with CD4+ and CD8+ T cell infiltration↓	(100)
miR-27a-3p	exosome	↑	MAGI2↓/PTEN↓/PI3K-AKT↑/PD-L1↑	BRCA	↓	immune evasion↑	(88)
miR-21	cancer cell	↑	PDCD4↓/PI3K/Akt/PD-L1↑	BRCA	↓	tolerance to radiotherapy and tumor immune escape↑	(89)
miR-1184	cancer cell	↓	circ0001598↑/miR-1184↓/PD-L1↑	BRCA	↓	tumor cell growth and trastuzumab-resistance↑	(90)
miR-3609	cancer cell	↓	PD-L1↑	BRCA	↓	sensitivity of tumor cells to adriamycin↓	(91)
miR-130a/145	Gr-1+CD11b+ immature myeloid cell	↓	TβRII↑/IGF1R↑	BRCA	↓	cytotoxic CD8+ T cells generated by IFNγ↓	(101)
hsa-miR-210-3p/190b	cancer cell	↑/↓	lnc00899/PSMG3-AS1/PAXIP1-AS1-has-miR-210-3p-CPEB2 SNHG16-hsa-miR-190b-BCL11A ceRNA	BRCA	↓	miR-210 promotes cancer growth through up-regulation, while miR-190b is anticarcinogenic by mediating apoptosis. CPEB2 functions as a tumor suppressor gene in breast cancer, while BCL11A is considered an oncogene in several cancers	(108)
let-7s	exosome	↓	Lin28B↑/let-7s↓/PD-L2↑	TNBC	↓	poor prognosis and lung metastasis↑	(92)
miR-622	cancer cell	↓	circ0000512↑/miR-622↓/CMTM6↑/PD-L1↑	TNBC	↓	TNBC progression and immune escape↑	(93)
cirmiR-20a-5p	exosome	↑	NPAT↓	TNBC	↓	resistance to anti-PD-1 immunotherapy↑	(94)
miR-145	cancer cell	↓	Cisplatin/miR-145↓/c-Myc/PD-L1↑	OV	↓	cisplatin resistance↑	(95)
miR-155-5p	exosome	↓	ROS/miR-155-5p↓/PD-L1↑	OV	↓	formation of an immunosuppressive TME↑	(16, 96)
miR-101-3p	EVs	↓	NEAT1↑/miR-101-3p↓/ZEB1↑/PD-L1↑	OV	↓	immune escape of cancer cells↑	(97)
miR-424 (322)	cancer cell	↑	PD-L1/PD-1↓ CD80/CTLA-4↓	OV	↑	MDSCs and Tregs↓	(98)
miR-20a	Serum	↑	NKG2D-MICA/B↓	OV	↓	tumor-killing effect of NK cells↓	(103)
miR-146a	cancer cell	↑	NF-κB↓/CXCL1↓	HGSC	↑	immune suppressive neutrophils↓	(107)
miR-124-3p	cancer cell	↓	lnc00240↑/miR-124-3p↓/pSTAT3↑/MICA↑	CESC	↓	Tumor growth, migration and invasion and NKT cell tolerance↑	(104)
miR-142-5p	exosome	↑	ARID2↓/DNMT1↓/IFN-γ↑/IDO↑	CESC	↓	suppress and exhaust CD8+ T cells↑	(102)
miR-421	cancer cell	↑	ACOXL-AS1↑/miR-421↑/MTF1↓	EEA	↓	respond to or escape from immunotherapy↑	(109)
miR-765	exosome	↓	ERβ↑/miR-765↓/PLP2↑/Notch↑	UCEC	↓	the proliferation and EMT of UCEC↑	(12)
miR-1245	NK cell	↑	NKG2D↓	HPV-induced cancer	↓	cancer immunosurveillance↓ and susceptibility to various malignancies↑	(105)

This table summarizes the role of various microRNAs (miRNAs) in regulating CD8^+^ T cell function and promoting immune evasion across multiple cancer types. It details how miRNAs from diverse cellular sources modulate key immune checkpoints (e.g., PD-L1), T cell activity, and cytokine secretion, leading to immunosuppression and resistance to therapies like anti-PD-1 treatment. The data underscore the critical function of miRNAs in shaping the tumor immune microenvironment.

BRCA, breast cancer; TNBC, triple-negative breast cancer; OV, ovarian cancer; HGSC, high-grade serous ovarian carcinoma; CESC, cervical squamous cell carcinoma; EEA, endometrioid endometrial adenocarcinoma; UCEC, uterine corpus endometrial carcinoma; HPV, human papillomavirus-induced cancers. Expression column: ↑, overexpression (upregulation); ↓, downregulation (suppression of expression). CD8^+^ T cell column: ↑, change that confers a benefit to CD8^+^ T cells (e.g., enhanced function, survival, or proliferation); ↓, change that is harmful to CD8^+^ T cells (e.g., impaired function or reduced viability).

#### 2.3.1 The PD-L1/PD-L2 immune checkpoint pathway and treatment resistance

Activation of the PD-L1 and PD-L2 pathways is a dominant mechanism across gynecological cancers, frequently driving resistance to various therapies. In breast cancer (BRCA), exosome miR-27a-3p upregulates PD-L1 through the MAGI2-PTEN-PI3K/AKT axis (88); miR-21 promotes PD-L1 expression via the PDCD4-PI3K/AKT axis, leading to radiation therapy resistance (89); downregulation of miR-1184 induces trastuzumab resistance via the circ0001598-PD-L1 axis (90); and the downregulation of miR-3609 directly promotes PD-L1 overexpression (91). In triple-negative breast cancer (TNBC), this axis is central to immune therapy resistance: downregulation of exosomes let-7s promotes PD-L2 overexpression via the Lin28B-PD-L2 axis, leading to lung metastasis and poor prognosis (92); downregulation of miR-622 drives immune escape through the circ0000512-CMTM6-PD-L1 cascade (93); and overexpression of exosome cirmiR-20a-5p targets NPAT to directly mediate resistance to anti-PD-1 therapy (94). Ovarian cancer also heavily relies on this pathway: downregulation of miR-145 directly mediates cisplatin resistance through c-Myc-induced PD-L1 overexpression (95); downregulation of miR-155-5p promotes an immunosuppressive microenvironment via the ROS-PD-L1 axis (96); and lncRNA NEAT1-regulated miR-101-3p drives immune escape through the ZEB1-PD-L1 cascade (97). Conversely, miR-424 (322) indirectly enhances antitumor immunity by inhibiting the PD-L1/CD80-CTLA-4 pathway (98).

#### 2.3.2 T cell dysfunction and exhaustion

miRNAs directly impair T cell functionality and promote immune exhaustion. In BRCA, the downregulation of miR-149-3p in CD8^+^ T cells induces the overexpression of inhibitory receptors (PD-1/TIM-3/BTLA), thereby inhibiting the secretion of crucial cytokines like IL-2, TNF-α, and IFN-γ (99). Additionally, abnormal expression of miR-34a/miR-494-3p reduces overall T cell infiltration (100), and the downregulation of miR-130a/145 in immature myeloid cells inhibits IFNγ-driven CD8^+^ T cell cytotoxicity through TβRII/IGF1R (101). Cervical cancer immune escape is significantly driven by T cell exhaustion, where the overexpression of exosome-derived miR-142-5p activates the IFNγ-IDO pathway by inhibiting ARID2/DNMT1, ultimately leading to CD8^+^ T cell exhaustion (102).

#### 2.3.3 Regulation of NK and NKT cell activity

Natural killer (NK) and natural killer T (NKT) cell tumor-killing capabilities are profoundly affected by miRNA dysregulation. In ovarian cancer, the overexpression of serum miR-20a downregulates NK cell activation ligands MICA/B, significantly impairing their tumor-killing ability (103). In cervical cancer, the downregulation of miR-124-3p in cancer cells upregulates MICA expression via the lnc00240-pSTAT3 axis, inducing NKT cell tolerance and promoting tumor growth and invasion (104). Furthermore, in HPV-related cancers, the overexpression of miR-1245 in NK cells downregulates NKG2D, thereby weakening tumor immune surveillance (105).

#### 2.3.4 Modulation of Tregs, suppressor cells, and TME remodeling

miRNAs reshape the TME by modulating immunosuppressive cells and facilitating broad survival mechanisms. Interestingly, some miRNAs exhibit anti-tumor regulatory effects: in BRCA, miR-126 is overexpressed in Tregs and inhibits Foxp3 through the p85β-PI3K/Akt axis, which actually weakens the immunosuppressive function of Tregs (106). In ovarian cancer, miR-424 (322) reduces the expansion of myeloid-derived suppressor cells (MDSCs) and Tregs (98). Furthermore, in high-grade serous carcinoma (HGSC), overexpression of miR-146a reduces immunosuppressive neutrophil infiltration by inhibiting NF-κB/CXCL1 signaling, further reshaping the TME (107). Other TME remodeling mechanisms involve complex ceRNA networks in BRCA, where miR-210-3p targets CPEB2 to promote cancer, while miR-190b exerts a tumor suppressor effect via BCL11A-induced apoptosis (108). In endometrial cancers, specific non-coding RNA pathways drive progression: endometrial adenocarcinoma (EEA) promotes immune therapy escape via the miR-421-ACOXL-AS1-MTF1 axis (109), while uterine corpus cancer (UCEC) relies on the downregulation of exosome miR-765 to activate epithelial-mesenchymal transition (EMT) through the ERβ-PLP2-Notch pathway, driving tumor proliferation and malignant progression (12).

### 2.4 Circulatory system and nervous system

The miRNA regulatory mechanisms of hematological and neurological tumors exhibit system-specific immune escape patterns, yet they share foundational pathways. In hematological tumors (such as leukemia and lymphoma) and nervous system tumors (such as glioblastoma), immune suppression is primarily achieved through a dynamic balance of core mechanisms: the regulation of immune checkpoints, T cell intrinsic dysfunction, metabolic reprogramming, and the remodeling of the TME. The precise regulation of these pro-inflammatory and anti-inflammatory miRNAs ultimately determines tumor progression. **Table 4** presents information on miRNAs related to circulatory tumors and neurological tumors mentioned above.

**Table 4:** miRNA-mediated regulation of CD8^+^ T cell function in hematological tumors and neurological tumors.

MicroRNA	Source	Express	Pathway	Cancer	CD8+T cell	Result	Ref.
miR-150	CD8+ T cell	–	miR-150(KO)/TMEM20↑/Ca2+↑/NFAT1↑/Cbl-b, Egr2, p27↑	AL	↓	anergy-promoting molecular milieu and function↑	(114)
miR-625-3p	CD8+ T cell	↑	CD3-TCR complex↑/miR-625-3p↑/IFN-γ↑	AL	↑	Proliferation level of CD8+ T cells after Allo-HSCT↑	(115)
miR-19a-3p	sEVs→ CD8+ T cell	–	NPM1/CTCF/PABPC1/miR-19a-3p/SLC6A8↓	LAML	↓	ATP production↓ and immune escape by leukemic cells↑	(120)
miR-142	myelocyte	mutate	wild-type: mTORC1↑ mutant -type: Akt-mTOR↓	LAML	↓	expression of distinct target genes↓	(121)
miR-20a-5p	CD3+ T cell	↓	NFAT2↓/miR-20a-5p↓	CML	↓	overall survival in patients↓	(86)
miR-195	cancer cell	↓	MALAT1↑/miR-195↓/PD-L1↑	DLBC	↓	proliferation, apoptosis and migration and immune escape abilities of DLBCL↑	(110)
miR-155	Serum	↑	pAKT-ERK↑/PD-L1↑	DLBC	↓	poor disease outcome↑	(111)
miR-340-5p	cancer cell/CD8+ T cell	↓	KMT5A↑/COP1/CD73↓	DLBC	↓	CD8+ T cell infiltration and antitumor function↓	(10)
miR-181a	cancer cell	↑	SOCS3↓/STAT3↑ DUSP6↓/pERK1/2↑	T-LGLL	↓	sensitivity to FAS-mediated apoptosis↓	(117)
miR-155/130/21	cancer cell	↑	pSTAT3↑/SOCS1, SOCS2, SOCS4↓/PD-L1, TIM3, LAG3, ICOS, CTLA4↑	CTCL	↓	CD8+ T-cell-mediated cytotoxic activity, with concordant production of IFN-γ and CD107a expression↓	(9)
miR-155/146a	CD4+/CD8+ T cell	–	miR-155 (KO)/Ship1↑/IFNγ↓ miR-146a(KO)/Irak1, Traf6↓/IFNγ↑	Lymphoma/SKCM	↓ ↑	miR-155 plays a dominant role in promoting the development of IFNγ-expressing T cells compared to miR-146a	(118)
miR-222/339	cancer cell	↑	Dicer/miR-222 and -339↑/ICAM-1↓	GBM	↓	cytolysis of tumor cells↓	(122)
miR-92a	cancer cell	↑	IL-6 and IL-10↑/IL-6(+) IL-10(+) NKT cell↑	GBM	↓	expression of anti-tumor molecules such as perforin, Fas ligand and interferon-γ in NKT cells↓	(123)
miR-15a/16	cancer cell	↑	mTOR/PD-1, Tim-3, LAG-3↑ and IFN-γ, IL-2, TNF-α↓	GBM	↓	Exhaustion↑ and activation↓ of glioma-infiltrating CD8+ T cells	(112)
miR-17-92	CD8+ T cell	↑	IFN-γ↑ and TGFBR2↓	GBM	↑	type-1 T-cell skewing and antitumor immunity↑ resistance to immunosuppressive effects of TGF-β↑	(124)
miR-326	cancer cell	↑	SMO-Gli2↓/TGF-β1↓	GBM	↑	immunosuppressive environment↓	(125)
miR-155	CD8+ T cell	–	miR-155(KO)/FoxO3a↑/Akt a, Stat5↓	GBM	↓	proliferative and invasive activities of T cell↓	(119)
miR-6794-5p	exosome	↑	SOCS1↓ JAK1-STAT3↑	GBM/LUAD	↓	M1 macrophages↓ and M2 macrophages↑ IL-10 secreted from M2 macrophages↑	(27)
miR-574-3p	cancer cell	↓	PD-L1↑	spinal chordoma	↓	TILs CD8+/TILs Foxp3+ ratio↑	(113)

This table summarizes the role of various microRNAs in regulating CD8^+^ T cell function in hematological malignancies (e.g., AL, LAML, DLBC) and nervous system tumors (e.g., GBM). It highlights how miRNAs from tumor cells and T cells modulate key pathways—including immune checkpoints, T cell metabolism, and cytokine signaling—to either promote immune evasion or enhance anti-tumor activity. These findings underscore the potential of miRNAs as therapeutic targets across diverse cancer types.

AL, acute leukemia; LAML, acute myeloid leukemia; CML, chronic myeloid leukemia; DLBC, diffuse large B-cell lymphoma; T-LGLL, T-cell large granular lymphocytic leukemia; CTCL, cutaneous T-cell lymphoma; SKCM, melanoma; GBM, glioblastoma; LUAD, lung adenocarcinoma. Expression column: ↑, overexpression (upregulation); ↓, downregulation (suppression of expression). CD8^+^ T cell column: ↑, change that confers a benefit to CD8^+^ T cells (e.g., enhanced function, survival, or proliferation); ↓, change that is harmful to CD8^+^ T cells (e.g., impaired function or reduced viability).

#### 2.4.1 The PD-L1 pathway and immune checkpoint regulation

Activation of PD-L1 and other inhibitory receptors is a dominant strategy across both circulatory and nervous system tumors. In lymphoma, this pathway is highly critical: in diffuse large B-cell lymphoma (DLBC), the downregulation of miR-195 enhances tumor proliferation, migration, and immune escape through the MALAT1-PD-L1 axis (110), while serum miR-155 overexpression predicts poor prognosis via the pAKT-ERK-PD-L1 axis (111). Cutaneous T-cell lymphoma (CTCL) heavily relies on the miR-155/130/21 cluster to upregulate inhibitory molecules such as PD-L1, TIM3, and LAG3 via the pSTAT3-SOCS1/2/4↓ axis, significantly impairing CD8^+^ T-cell cytotoxicity (9). Similar checkpoint exploitation is observed in nervous system tumors: in glioblastoma (GBM), miR-15a/16 upregulation induces the overexpression of immune checkpoint molecules (PD-1, Tim-3, and LAG3) through the activation of the mTOR signaling axis, ultimately driving CD8^+^ T cell exhaustion (112). Additionally, in chordomas, the downregulation of miR-574-3p induces PD-L1 overexpression, which leads to a reduced ratio of CD8^+^ T cells to Tregs, further exacerbating immunosuppression (113).

#### 2.4.2 T cell dysfunction, exhaustion, and apoptosis

miRNAs directly modulate T cell activity by interfering with TCR signaling, calcium cascades, and cellular survival. In acute leukemia (AL), this regulation is bidirectional: miR-150 deficiency upregulates Cbl-b/Egr2/p27 through the TMEM20-Ca²^+^-NFAT1 axis, inducing CD8^+^ T cell unresponsiveness (114); conversely, the upregulation of miR-625-3p enhances CD3-TCR signaling, promoting CD8^+^ T cell proliferation and IFN-γ secretion following allogeneic hematopoietic stem cell transplantation (115). In chronic myeloid leukemia (CML), the downregulation of miR-20a-5p in CD3^+^ T cells reduces NFAT2 expression, directly correlating with decreased overall patient survival (116). Lymphomas also utilize direct T cell modulation: in T-LGL leukemia, miR-181a overexpression reduces FAS-mediated apoptosis sensitivity through dual mechanisms involving SOCS3/STAT3 and DUSP6/pERK1/2 (117). Cross-lymphoma studies further highlight this by revealing that miR-155 knockout inhibits T cell function (Ship1↑/IFNγ↓), whereas miR-146a knockout enhances antitumor immunity (Irak1/Traf6↓/IFNγ↑) (118). In GBM, the loss of miR-155 significantly inhibits T cell proliferation by upregulating FoxO3a and inhibiting the Akt-Stat5 pathway (119).

#### 2.4.3 Metabolic reprogramming and infiltration barriers

Metabolic manipulation is a core driver of immune escape, particularly in leukemias. In acute myeloid leukemia (LAML), small extracellular vesicles (sEVs) derived from leukemia cells deliver miR-19a-3p to CD8^+^ T cells, which inhibits SLC6A8 and reduces ATP production, thereby driving immune escape (120). Furthermore, myeloid cell miR-142 mutants downregulate target genes via Akt-mTOR signaling, while wild-type miR-142 activates the mTORC1 pathway, altering the cellular metabolic state (121). Beyond intrinsic metabolism, miRNAs also create metabolic and physical barriers to infiltration; for example, in DLBC, miR-340-5p downregulation inhibits CD8^+^ T cell infiltration and antitumor function through the KMT5A-COP1/CD73 axis (10).

#### 2.4.4 Tumor microenvironment remodeling and cytokine regulation

The TME exhibits unique dynamic equilibrium characteristics, where the balance of pro-inflammatory and anti-inflammatory miRNAs dictates tumor progression. In GBM, tumors evade immunity by upregulating specific suppressive miRNAs: overexpression of miR-222/339 downregulates ICAM-1 expression, effectively inhibiting tumor cell lysis (122); miR-92a upregulation promotes the expansion of IL-6/IL-10-positive NKT cells while reducing perforin and IFN-γ levels (123); and tumor-derived exosome (TEX) miR-6794-5p drives M2 macrophage polarization and enhances IL-10 secretion by downregulating SOCS1 and activating JAK1-STAT3 signaling (27). Conversely, the expression of certain miRNAs actively enhances anti-tumor responses: the miR-17–92 cluster in CD8^+^ T cells resists TGF-β-mediated immunosuppression by upregulating IFN-γ and downregulating TGFBR2 (124), while miR-326 overexpression reverses the immunosuppressive microenvironment by inhibiting the SMO-Gli2/TGF-β1 signaling pathway (125). The complex interaction of pro-inflammatory (e.g., miR-17-92, miR-326) and anti-inflammatory (e.g., miR-92a, miR-15a/16) miRNAs highlights the precision of TME regulation in neurological tumors.

### 2.5 Urinary system, head and neck, endocrine system, musculoskeletal system

Although tumors of the urinary tract, head and neck, endocrine system, and musculoskeletal system originate from vastly different anatomical sites, their miRNA-mediated immune escape strategies converge on several shared mechanistic hubs. Instead of acting in isolation, these non-coding RNAs construct complex, cross-system regulatory networks. The formation of the immunosuppressive microenvironment in these diverse malignancies is primarily driven by the synergistic interaction of immune checkpoint regulatory networks, metabolic reprogramming, direct apoptosis regulation, and metastasis signaling. **Table 5** demonstrates the diverse mechanisms exhibited by these miRNAs across different tumor types.

**Table 5:** miRNA-mediated regulation of CD8^+^ T cell function in urinary system tumors, head and neck tumors, endocrine system tumors, musculoskeletal system tumors.

MicroRNA	Source	Express	Pathway	Cancer	CD8+T cell	Result	Ref.
miR-29b/198	CD8+ T cell	↑	JAK↓/MCL-1↓	KIRC	↓	levels of anti-apoptosis and proliferation-related gene products expressed by CD8+ T cells↓	(134)
miR-195/-16	cancer cell	↓	PD-L1/PD-1↑ CD80/CTLA-4↑	PRAD	↓	MDSCs and Tregs↑	(126)
miR-105-5p	cancer cell/CD8+ T cell	↓	lnc00184↑/miR-105-5p↓/PD-L1↑	PRAD	↓	docetaxel resistance and immune escape in cancer cells↑	(127)
miR-141-3p	cancer cell	↓	circTRPS1↑/miR141-3p↓/GLS1↑	BLCA	↓	intracellular ROS balance↓	(131)
miR-320a/301b-3p	PMN-MDSC	↓	circ0013936↑/miR-320a↓/JAK2↑/FATP2↑ circ0013936↑/miR-301b-3p↓/CREB1↑/RIPK3↓	BLCA	↓	immunosuppressive activity of PMN-MDSCs↑	(132)
miR-206	cancer cell	↓	lnc00460↑/miR-206↓/STC2, AKT, ERK↑	HNSCC	↓	apoptosis and autophagy of HNSCC cells↑	(135)
miR-214-3p	cancer cell	↓	lnc01123↑/miR-214-3p↓/B7-H3↑	HNSCC	↓	tumorigenicity of HNSCC cells↑and secretion of immune-related factors *in vivo*↓	(129)
miR-21	exosome	↑	Snail↑/miR-21↑/PTEN, BRCC3↓/pNLRP3↑	HNSCC	↓	inflammatory vesicle activity of tumor-associated TAMs↓	(133)
miR-375	CSC	↓	PVT1↑/miR-375↓/YAP1↑	HNSCC	↓	CSCs and metastasis↑	(137)
miR-495-3p	cancer cell	↓	circKRT1↑/miR-495-3p↓/PD-L1↑	OSCC	↓	cancer progression and immune evasion↑	(128)
miR-148	cancer cell	↓	UCA1↑/miR-148a↓/PD-L1↑	THCA	↓	the killing effect of cytotoxic CD8+ T cells and reduced cytokine secretion↓	(69)
miR-519e-5p	exosome	↑	hnRNPA2B1/miR-519e-5p↑/PLAGL2↑/Wnt↑/NOTCH2↓	PTC	↓	tumor cell dissemination and metastasis↑	(128)
miR-200a	cancer cell	↑	PTEN↓/PD-L1↑	OS	↓	percentage of Foxp3+ Tregs↑	(130)

This table summarizes miRNA-mediated regulation of CD8^+^ T cell function in urological (KIRC, BLCA, PRAD), head and neck (HNSCC, OSCC), endocrine (THCA, PTC), and musculoskeletal (OS) tumors. It details how miRNAs from tumor cells and immune cells modulate key pathways—particularly the PD-L1 axis and other immune checkpoints—to promote an immunosuppressive microenvironment, drive immune evasion, and impact response to therapy.

KIRC, kidney renal clear cell carcinoma; PRAD, prostate adenocarcinoma; BLCA, bladder urothelial carcinoma; HNSCC, head and neck squamous cell carcinoma; OSCC, oral squamous cell carcinoma; THCA, thyroid carcinoma; PTC, papillary thyroid carcinoma; OS, osteosarcoma. Expression column: ↑, overexpression (upregulation); ↓, downregulation (suppression of expression). CD8^+^ T cell column: ↑, change that confers a benefit to CD8^+^ T cells (e.g., enhanced function, survival, or proliferation); ↓, change that is harmful to CD8^+^ T cells (e.g., impaired function or reduced viability).

#### 2.5.1 The PD-L1 and immune checkpoint regulatory network

Activation of PD-L1 and other immune checkpoints via non-coding RNA axes is a dominant immune evasion strategy across these diverse tumors. In prostate adenocarcinoma (PRAD), the downregulation of miR-195/16 promotes the expansion of myeloid-derived suppressor cells (MDSCs) and Tregs through the PD-L1/PD-1 and CD80/CTLA-4 axes (126), while miR-105-5p downregulates docetaxel resistance and immune escape via the lnc00184-PD-L1 cascade (127). Head and neck tumors also heavily rely on this mechanism; in oral squamous cell carcinoma (OSCC), the downregulation of miR-495-3p drives tumor progression and immune escape through the circKRT1-PD-L1 axis (128). In head and neck squamous cell carcinoma (HNSCC), the downregulation of miR-214-3p not only enhances tumorigenesis via the lnc01123-B7-H3 axis but also significantly inhibits the secretion of immune factors (129). Furthermore, endocrine and musculoskeletal tumors utilize this pathway: in thyroid cancer (THCA), the downregulation of miR-148 through the lncRNA UCA1-PD-L1 axis inhibits the cytotoxic activity and cytokine secretion of CD8^+^ T cells (85), while in osteosarcoma (OS), miR-200a upregulation increases the proportion of Foxp3^+^ Tregs through the PTEN↓-PD-L1↑ axis (130).

#### 2.5.2 Metabolic reprogramming and suppressor cell expansion

Metabolic imbalance and the recruitment of immunosuppressive cells form another core feature of the TME in these systems. In bladder cancer (BLCA), miR-141-3p downregulates the circTRPS1-GLS1 axis to disrupt the balance of reactive oxygen species (ROS) in cells (131). Furthermore, the downregulation of miR-320a/301b-3p in polymorphonuclear MDSCs enhances their immunosuppressive activity through the circ0013936-JAK2/FATP2↑ and CREB1↑/RIPK3↓ axes (132). In HNSCC, exosome-derived miR-21 upregulation inhibits tumor-associated macrophage (TAM) inflammasome activity through Snail-mediated PTEN/BRCC3 downregulation and pNLRP3 upregulation, thereby weakening the anti-tumor immune response (133).

#### 2.5.3 Direct regulation of apoptosis and cell survival

miRNAs directly modulate the survival of both T cells and tumor cells to tip the balance in favor of cancer progression. In clear cell renal cell carcinoma (KIRC), the upregulation of miR-29b/198 in CD8^+^ T cells downregulates anti-apoptotic genes through the JAK-MCL-1 axis, fundamentally impairing T cell function and survival (134). Conversely, in HNSCC, the downregulation of miR-206 protects tumor cells by effectively inhibiting apoptosis and autophagy through the activation of the lnc00460-STC2/AKT/ERK signaling axis (135).

#### 2.5.4 Tumor metastasis and stemness maintenance

Beyond direct immune suppression, miRNAs facilitate systemic tumor progression by promoting metastasis and preserving cancer stem cell (CSC) traits. In papillary thyroid carcinoma (PTC), tumor dissemination and metastasis are promoted through the upregulation of exosome miR-519e-5p and the imbalance of the hnRNPA2B1-PLAGL2-Wnt/NOTCH2 axis (136). Similarly, in HNSCC cancer stem cells, miR-375 downregulation promotes stemness maintenance and metastatic ability through the PVT1-YAP1 axis (137).

### 2.6 Melanoma

In typical experimental models of melanoma, various miRNAs profoundly influence the effectiveness of tumor immune responses through precise regulation of immune cell functions, signaling pathways, and the tumor microenvironment (TME). Instead of acting in isolation, these non-coding RNAs form a highly complex and bidirectional regulatory network. The mechanisms dictating melanoma immune evasion or anti-tumor immunity can be categorized into three primary functional axes: hypoxia-induced immune escape, the intrinsic enhancement of T cell function and memory, and the bidirectional modulation of innate and adaptive immunity. **Table 6** elaborates in detail the complex mechanism of action of miRNAs in the immune regulation of melanoma.

**Table 6:** miRNA-mediated regulation of CD8^+^ T cell function in melanoma.

MicroRNA	Source	Express	Pathway	Cancer	CD8+T cell	Result	Ref.
miR-210	Hypoxic cancer cell	↑	PTPN1, HOXA1, TP53I11↓	NSCLC/SKCM	↓	resistance in hypoxic tumor targets to CD8+ T cell -mediated lysiss↑	(24)
miR-192-5p	hypoxic cancer cell	↑	Cx43-GJs/miR-192-5p↑	SKCM	↓	ZEB2 mRNA expression↓	(138)
hsa-miR-122/149/498/3187-3p/181	exosome	↑	hsa-miR-498/TNFα↓ hsa-miR-181/TNFα↓ hsa-miR-3187-3p/CD45↓/TNFα↓	SKCM	↓	tumor immune evasion↑	(140)
miR-34a	cancer cell	↓	KCNQ1OT1↑/miR-34a↓/pSTAT3↑/PD-L1↑	SKCM	↓	proliferation, migration, and invasion of melanoma cells↑	(139)
miR-155	CD8+ T cell	↑	SOCS-1↓/STAT5↑	SKCM	↑	immunotherapies for cancer↑	(141)
miR-155	CD8+ T cell	↑	Ship1↓/Akt↑ Socs1, Ptpn2↓/Stat5↑	SKCM	↑	effectiveness of adoptive immunotherapies in a cell-intrinsic manner↑	(142)
miR-155	CD8+ T cell	↑	NFκB, AP-1, IRF4↑/miR-155↑/PD-1↓/TCR, CD28↓	SKCM	↑	frequencies in tumor-infiltrated lymph nodes↑	(143)
miR-155	CD8+ T cell	↑	Ship1↓/Phf19↑/PRC2↑	SKCM	↑	T cell senescence↓ and sustain CD8+ T cell antitumor responses↑	(145)
miR-155	OT-1 CD8+ T cell	↑	INPP5D↓/pAkt↑/mTOR↑	SKCM	↑	CD8 downregulation occurs during T cell activation↓	(144)
hsa-miR-320a-3p/mmu-miR-7037-5p/666-3p	cancer cell	↑	Psmc3, Ndufa1	SKCM	↑	the susceptibility of melanoma cells to T cell-mediated killing↑	(148)
miR-25-3p/155-5p/215-5p/375	EVs	↑	CD4+T cell→EVs→CD8+ T cell	SKCM	↑	CD8+ T cell-mediated antitumor responses↑	(147)
let-7	CD8+ T cell	↑	PI3K/AKT/mTOR↓ ROS↓	SKCM	↑	time-sensitive support of memory formation and protection of effector cells from exhaustion↑	(146)
miR-155/146a	CD4+/CD8+ T cell	–	miR-155 (KO)/Ship1↑/IFNγ↓ miR-146a(KO)/Irak1, Traf6↓/IFNγ↑	Lymphoma/SKCM	↓ ↑	miR-155 plays a dominant role in promoting the development of IFNγ-expressing T cells compared to miR-146a	(118)
miR-23a/24a/27a	NK cell	↓	Mirc11(KO)/A20, Itch, Cbl-b↑/K63, TRAF6↓/NF-Κb, AP-1↑	SKCM	↓	NK cell-mediated proinflammatory response↓ generation of proinflammatory factors *in vitro*↓	(149)

This table summarizes miRNA-mediated regulation of CD8^+^ T cell function in melanoma. It details how miRNAs from tumor cells and immune cells modulate key pathways—particularly the PD-L1 axis and other immune checkpoints—to promote an immunosuppressive microenvironment, drive immune evasion, and impact response to therapy. Expression column: ↑, overexpression (upregulation); ↓, downregulation (suppression of expression). CD8^+^ T cell column: ↑, change that confers a benefit to CD8^+^ T cells (e.g., enhanced function, survival, or proliferation); ↓, change that is harmful to CD8^+^ T cells (e.g., impaired function or reduced viability).

#### 2.6.1 Hypoxia-induced escape and immune checkpoint regulation

The hypoxic microenvironment of melanoma serves as a major catalyst for miRNA-driven immune suppression. Under hypoxic conditions, elevated expression of miR-210 in tumor cells directly reduces CD8^+^ T cell activity and enhances tumor resistance to T cell-mediated lysis through the inhibition of PTPN1, HOXA1, and TP53I11 (24). Similarly, miR-192-5p is upregulated in hypoxic cancer cells, where it targets connexin-43 (Cx43)-constituted gap junctions (GJs) and reduces ZEB2 mRNA, further suppressing CD8^+^ T cell activity (138). Beyond hypoxia, the classic PD-L1 immune checkpoint pathway is actively manipulated: miR-34a is downregulated in cancer cells, which upregulates PD-L1 through the KCNQ1OT1/miR-34a/pSTAT3 axis, promoting melanoma proliferation, migration, and invasion while simultaneously inhibiting CD8^+^ T cell activity (139). Furthermore, intercellular communication via exosome-derived miRNA clusters (including elevated hsa-miR-122/149/498/3187-3p/181) plays a suppressive role; specifically, miR-498/181/3187-3p inhibit TNFα or CD45 to actively promote tumor immune escape (140).

#### 2.6.2 Intrinsic enhancement of T cell function and memory

Conversely, multiple miRNAs act as potent enhancers of anti-tumor immunity, primarily by optimizing T cell survival, metabolism, and cytotoxicity. miR-155 acts as a central hub in this process; when highly expressed in CD8^+^ T cells, it comprehensively enhances anti-tumor immune responses and improves immunotherapy efficacy through several simultaneous pathways. It activates STAT5 by inhibiting SOCS-1 (141), activates Akt/Stat5 by inhibiting Ship1 and Socs1/Ptpn2 (142), activates NFκB/AP-1/IRF4 while inhibiting PD-1 (143), and activates pAkt/mTOR by inhibiting INPP5D (144, 145). Furthermore, miR-155 delays T cell senescence by inhibiting Ship1 and upregulating Phf19/PRC2 (145).

Memory formation and intercellular support are also heavily regulated by miRNAs. The upregulation of let-7 in CD8^+^ T cells protects effector T cells from exhaustion and promotes memory formation by inhibiting the PI3K/AKT/mTOR pathway and reducing reactive oxygen species (ROS) (146). Additionally, extracellular vesicles (EVs) delivering miR-25-3p/155-5p/215-5p/375 from CD4^+^ T cells to CD8^+^ T cells significantly enhance the anti-tumor response of the recipient CD8^+^ T cells (147). Finally, the high expression of specific miRNA clusters in cancer cells, such as hsa-miR-320a-3p/mmu-miR-7037-5p/666-3p, targets Psmc3/Ndufa1, making the melanoma more sensitive to T cell-mediated killing (148).

#### 2.6.3 Bidirectional regulation and innate immune modulation

The miRNA network in melanoma exhibits remarkable bidirectionality and complexity. This is most evident in the opposing effects of miR-155 and miR-146a in CD4^+^/CD8^+^ T cells: while miR-155 deficiency inhibits IFNγ via the upregulation of Ship1, miR-146a deficiency actually enhances IFNγ through the inhibition of Irak1/Traf6. Ultimately, in melanoma (as in lymphoma), miR-155 plays the dominant role in promoting the development of IFNγ^+^ T cells (118). This complex regulation extends to the innate immune system as well. In natural killer (NK) cells, the downregulation of miR-23a/24a/27a leads to the upregulation of A20/Itch/Cbl-b and the inhibition of TRAF6/NF-κB/AP-1, fundamentally weakening the pro-inflammatory response and *in vitro* cytokine production of these crucial innate effector cells (149).

## 3 Potential drugs or treatment

### 3.1 Delivery system

**Figure 4:**
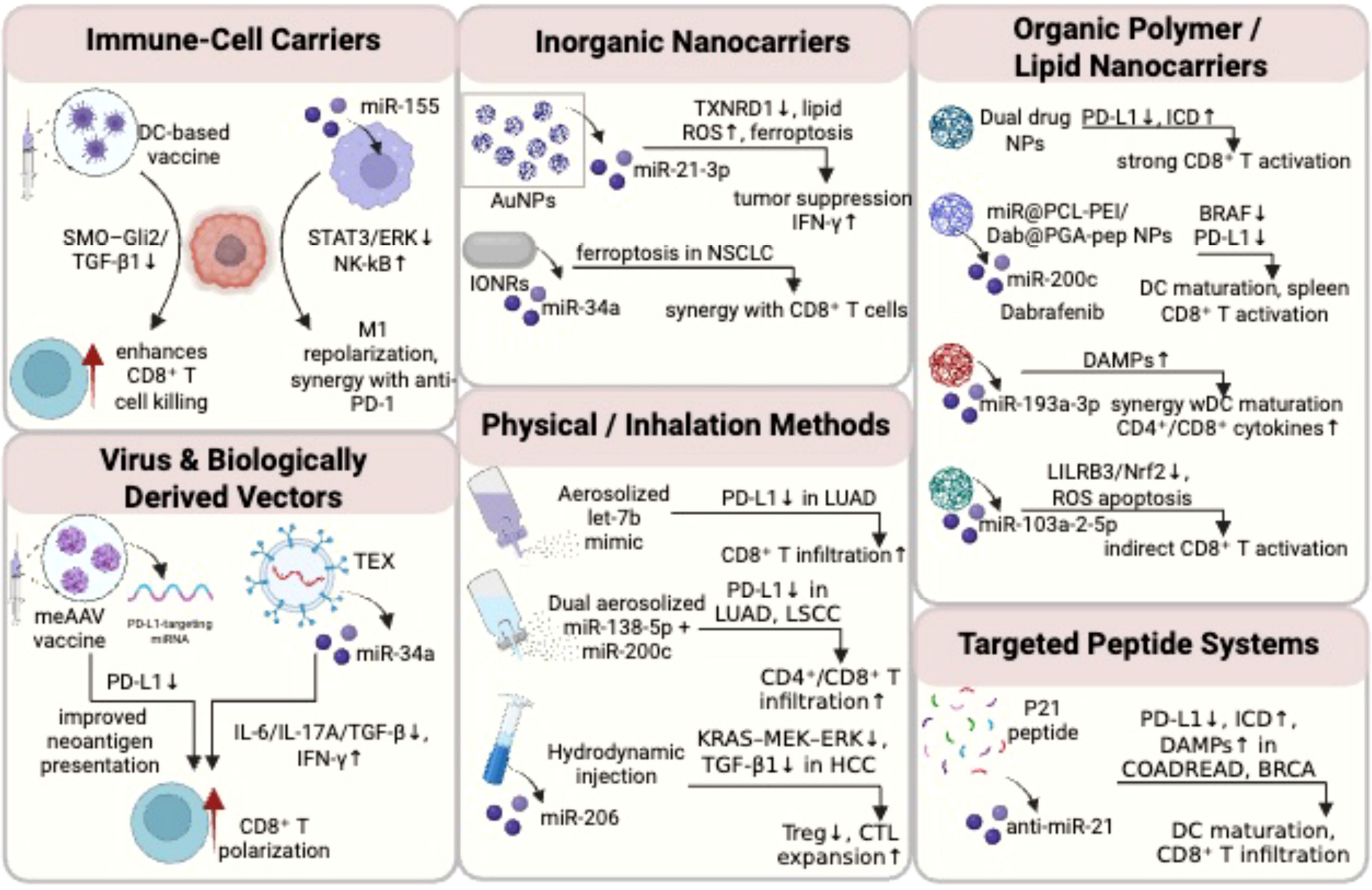
Diverse miRNA delivery systems for tumor immunotherapy: carrier platforms and immune regulatory mechanisms. Schematic summary of diverse delivery systems for microRNAs (miRNAs) in cancer immunotherapy. It categorizes strategies into immune-cell carriers, inorganic nanocarriers, organic polymer/lipid nanocarriers, virus & biologically derived vectors, physical/inhalation methods, and targeted peptide systems, each illustrating specific miRNAs, mechanisms, and effects on immune modulation (e.g., CD8^+^ T cell activation, PD-L1 regulation).

miRNA delivery systems have demonstrated significant potential in tumor immune therapy, with researchers developing various innovative carrier platforms to achieve efficient miRNA delivery and immune regulation. **Figure 4** presents diverse miRNA delivery systems for tumor immunotherapy, covering immune-cell carriers, inorganic nanocarriers, organic polymer/lipid nanocarriers, virus/biologically derived vectors, physical/inhalation methods, and targeted peptide systems. They deliver miRNAs to regulate immune responses (e.g., CD8^+^ T activation, PD-L1 modulation, macrophage repolarization), demonstrating significant potential in tumor immunotherapy.

In terms of immune cell carrier systems, dendritic cell (DC) vaccines have been successfully used to deliver miR-326, targeting GBM cells and inhibiting the SMO-Gli2/TGF-β1 pathway, effectively alleviating Treg-mediated immune suppression, thereby enhancing the specific cytotoxic activity of CD8^+^ T cells against U87-EGFRvIII tumors (125). Additionally, layered double hydroxides (LDHs) as a novel carrier can specifically deliver miR-155 to TAMs, inhibiting the STAT3/ERK pathway and activating the NF-κB signaling pathway, successfully driving TAMs toward M1-type repolarization, and synergistically enhancing the antitumor activity of CD8^+^ T cells with anti-PD-1 therapy (150).

Viruses and biologically derived vector systems also demonstrate unique advantages. Modified adeno-associated virus (meAAV) serves as a new antigen vaccine carrier. By carrying PD-L1-targeted miRNA, it can specifically silence PD-L1 expression in DCs, significantly enhancing new antigen presentation efficiency and activating tumor-specific CD8^+^ T cells. When combined with radiotherapy, it effectively eliminates COADREAD and BRCA (151). Meanwhile, TEX has been ingeniously modified to deliver miR-34a to T cells, successfully driving the polarization of CD8^+^ T cells in tumors, lymph nodes, and spleens by inhibiting IL-6/IL-17A/TGF-β and promoting IFN-γ secretion, thereby effectively inhibiting colorectal cancer growth (152).

In the field of inorganic nanocarriers, a gold nanoparticle delivery system delivers miR-21-3p to melanoma cells, inhibiting TXNRD1 expression and inducing the accumulation of lipid ROS and ferroptosis. This not only directly kills tumor cells but also enhances CD8^+^ T cell secretion of IFN-γ, producing a synergistic antitumor effect with anti-PD-1 therapy (153). Similarly, iron oxide nanorods (IONR) deliver miR-34a to NSCLC cells, inducing ferroptosis and synergizing with activated CD8^+^ T cells (Jurkat model) to achieve efficient tumor killing (154).

Organic polymer/lipid nanocarrier systems demonstrate outstanding performance in combination delivery strategies. PLGA-PEI/PLGA-PEG-FA dual-drug nanoparticles can simultaneously deliver doxorubicin (DOX) and miR-200c to cancer cells, effectively activating CD8^+^ T cells and significantly inhibiting tumor growth by suppressing PD-L1 expression and inducing immunogenic cell death (ICD) (155). The CXCR4-targeted nanoparticle system uses PGA-pep-modified PCL-PEI nanoparticles to co-deliver dabrafenib (a BRAF inhibitor) and miR-200c, inhibiting the PD-L1/BRAF signaling pathway, promoting DC maturation, and activating splenic CD8^+^ T cells, thereby effectively inhibiting melanoma progression (156). Lipid nanoparticles (LNPs) delivering miR-193a-3p can induce tumor cells to release damage-associated molecular patterns (DAMPs), promote DC maturation, and activate CD4^+^/CD8^+^ T cells to secrete type I cytokines, establishing long-term antitumor immune memory (157). Cationic liposomes (CLP) delivering miR-103a-2-5p to AML cells induce ROS-dependent apoptosis by inhibiting the LILRB3/Nrf2/HO-1 pathway, thereby indirectly activating CD8^+^ T cells (158). Additionally, rHDL-modified liposomes co-deliver α-Hederin and oxaliplatin (OXA) to colorectal cancer cells, activating CD8^+^ T cells by regulating the miR-140-5p/PRDM1 axis and inhibiting the KEAP1/NRF2 pathway to induce ferroptosis, successfully overcoming chemotherapy resistance (159).

Physical/inhalation delivery systems offer a unique approach for lung tumor therapy. Inhalation aerosol delivery of let-7b to lung cancer cells and CD8^+^ T cells inhibits the PD-L1/PD-1 pathway, reduces Tregs, and increases CD8^+^ T cell infiltration, effectively suppressing B[a]P-induced LUAD (12). A dual miRNA aerosol system co-delivers miR-138-5p and miR-200c, synergistically inhibiting PD-L1 expression and increasing CD4^+^/CD8^+^ T cell infiltration in lung tissue, successfully blocking the progression of LUAD and squamous cell carcinoma (LSCC) (160). Hydrodynamic injection (HDI) technology delivers miR-206 to hepatocellular carcinoma cells, inhibiting the KRAS-MEK-ERK/TGFβ1 pathway, reducing Treg expansion, and enhancing CTL toxicity, achieving 100% prevention of HCC (83).

Finally, multifunctional targeted peptide systems demonstrate potential for precision therapy. PD-L1-targeted peptides (P21) deliver anti-miR-21 to colorectal and breast cancer cells, inhibiting miR-21-induced ICD, releasing DAMPs to promote DC maturation and CD8^+^ T cell infiltration, and synergizing with anti-PD-L1 therapy to successfully eliminate primary and metastatic lesions (161). These diverse miRNA delivery systems provide a diverse technical platform for tumor immunotherapy, demonstrating broad clinical translation potential. **Table 7** details key info of 16 miRNA delivery systems (miRNA, action site, regulatory pathway, etc.). miRNA delivery systems have great tumor immunotherapy potential; researchers develop innovative carriers, e.g., DC vaccines deliver miR-326 to enhance CD8^+^T cells’ GBM-killing effect.

**Table 7:** Key information of miRNA delivery systems in tumors (miRNA, action location, regulatory pathway, targeted cancer, CD8^+^T cell changes and outcomes).

Deliver system	MicroRNA	Location	Pathway	Cancer	CD8+T cell	Result	Ref.
DC-based vaccine	miR-326	U87-EGFRvIII cell	SMO-Gli2↓/TGF-β1↓	GBM	↑	In terms of killing U87-EGFRvIII cells↑	(125)
LDHs	miR155	macrophage	STAT3/ERK1/2↓ NF-κB↑	–	↑	Synergistic repolarization of TAMs and α-PD-1 efficacy↑	(150)
meAAV neoAg vaccine	PD-L1-targeting miRNA	DC	PD-L1↓	COADREAD/BRCA	↑	neoAg presentation boost	(151)
TEX-miR-34a	miR-34a	T cell	IL-6、IL-17A、TGF-β↓ IFN-γ↑	COADREAD	↑	T cell polarization toward CD8+ T subsets among tumor-infiltrating lymphocytes, DLNs and spleen cells	(152)
gold NP	miR-21-3p	cancer cell	TXNRD1↓/Lipid ROS↑/IFN-γ↑	SKCM	↑	Synergistic tumor suppression with anti-PD-1	(153)
IONR	miR-34a	cancer cell	ferroptosis	NSCLC	↑	synergistic tumor cell death	(154)
dual drug-loaded NP	miR-200c	cancer cell	PD-L1↓	–	↑	Significant tumor growth inhibition	(155)
miR@PCL-PEI/Dab@PGA-pep nanoformulation	miR-200c	cancer cell	BRAF↓/PD-L1↓	SKCM	↑	the DC maturation in lymph node and CD8+ T cell activation in the spleen↑	(156)
LNP	miR-193a-3p(INT-1B3)	cancer cell	DAMPs↑→DC maturation→T-cell activation	–	↑	production of type 1 cytokines by CD4+ and CD8+ T cells↑	(157)
CLP	miR-103a-2-5p	cancer cell	LILRB3↓ Nrf2/HO-1↓	AML	↑	excessive intracellular ROS that may promote AML cell apoptosis	(158)
α-Hederin-OXA-rHDL	miR-140-5p	cancer cell	KEAP1, NRF2↓/HO1, GPX4↓	COADREAD	↑	oxaliplatin resistance↓	(159)
aerosolize	let-7b mimic	CD8+ T cell/cancer cell	PD-L1↓	B[a]P-induced LUAD	↑	tumor inhibition↑	(12)
aerosolize	miR-138-5p/miR-200c	lung tissue	PD-L1↓	B[a]P-induced LUAD and NTCU-induced LSCC	↑	CD4+ and CD8+ T cells↑	(160)
HDI	miR-206	cancer cell	KRAS-MEK-ERK↓/TGFβ1↓	HCC	↑	suppressor function and expansion of Tregs↓ expansion and cytotoxic program of CTLs↑	(83)
P21	miR-21	cancer cell	PD-L1↓	COADREAD/BRCA	↑	conferring immunogenicity to dying cancer cells and promoting dendritic cell maturation	(161)
NP	miR-146a	cancer cell	NF-κB↓/CXCL1↓	HGSC	↑	immune suppressive neutrophils ↓and CTL infiltration↑	(107)

This table summarizes key information on miRNA delivery systems in cancer therapy, including the delivery vehicle (e.g., nanoparticles, viral vectors), the specific miRNA delivered, its action location, the molecular pathway it regulates, the targeted cancer type, the resulting change in CD8^+^ T cell activity, and the therapeutic outcome. These systems are designed to modulate the tumor immune microenvironment, primarily by repolarizing immunosuppressive cells or downregulating checkpoint proteins like PD-L1, thereby enhancing CD8^+^ T cell function and achieving synergistic antitumor effects with existing immunotherapies.

DC, dendritic cell; LDHs, layered double hydroxides; meAAV neoAg vaccine, mutated engineered adeno-associated virus neoantigen vaccine; TEX, tumor-derived exosomes; gold NP, gold nanoparticles; IONR, iron oxide nanorods; dual drug-loaded NP, dual drug-loaded nanoparticles; miR@PCL-PEI/Dab@PGA-pep nanoformulation, poly(ϵ-caprolactone)-polyethylenimine/poly(glutamic acid)-peptide nanoformulation; LNP, lipid nanoparticles; CLP, cationic lipoplexes; α-Hederin-OXA-rHDL, α-hederin-oxaliplatin-reconstituted high-density lipoprotein; HDI, hydrodynamic injection; P21, polyplexes from self-assembling 21-mer polypeptide; NP, nanoparticles; GBM, glioblastoma; COADREAD, colorectal adenocarcinoma; BRCA, breast cancer; SKCM, skin cutaneous melanoma; NSCLC, non-small cell lung cancer; AML, acute myeloid leukemia; LUAD, lung adenocarcinoma; LSCC, lung squamous cell carcinoma; HCC, hepatocellular carcinoma; HGSC, high-grade serous ovarian carcinoma. CD8^+^ T cell column: ↑, change that confers a benefit to CD8^+^ T cells (e.g., enhanced function, survival, or proliferation); ↓, change that is harmful to CD8^+^ T cells (e.g., impaired function or reduced viability).

### 3.2 Drug

**Figure 5:**
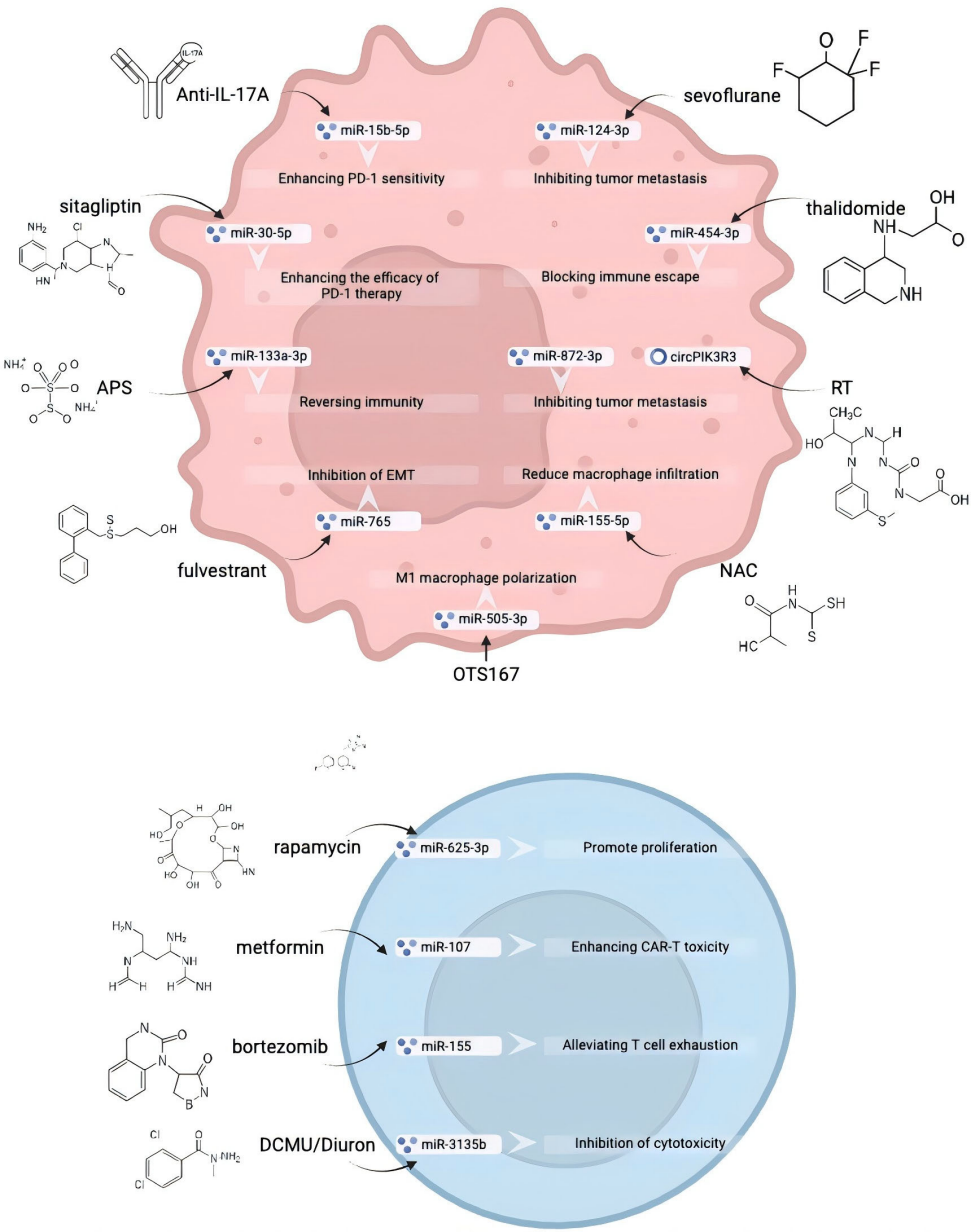
Drug-mediated miRNA regulation in cancer cells and CD8+ T cells for tumor immunotherapy. In cancer cells, drugs like sitagliptin, anti-IL-17A, and sevoflurane target miRNAs (e.g., miR-30-5p, miR-15b-5p, miR-124-3p) to enhance PD-1 therapy efficacy, inhibit metastasis, and block immune escape. In CD8^+^ T cells, rapamycin, metformin, and bortezomib regulate miRNAs (e.g., miR-625-3p, miR-107, miR-155) to promote proliferation, enhance CAR-T toxicity, and alleviate T cell exhaustion. These miRNA-mediated drug actions provide novel intervention strategies for tumor immunotherapy.

Multiple drugs can modulate CD8^+^ T cell function by regulating miRNA networks, offering new intervention strategies for tumor immunotherapy. In terms of immune checkpoint regulation, multiple drugs target the PD-1/PD-L1 pathway through different miRNA pathways, significantly enhancing the efficacy of immunotherapy. **Figure 5** illustrates how multiple drugs modulate CD8^+^ T cell function and cancer cell behavior via miRNA regulation in tumor immunotherapy. Sitagliptin targets the DPP4/CXCL10 axis by downregulating miR-30-5p in hepatocellular carcinoma, effectively enhancing CD8^+^ T cell infiltration and improving the efficacy of anti-PD-1 therapy (72). Anti-IL-17A antibodies block the IL-17A/P65/NRF1 axis to upregulate miR-15b-5p, thereby not only inhibiting PD-L1 expression in colorectal cancer but also reducing MDSC infiltration, which significantly enhances the susceptibility to PD-1 inhibitors (49). Metformin downregulates miR-107 by activating AMPK, thereby increasing Eomes expression and inhibiting PD-1, successfully promoting CAR-T cell cytotoxicity and memory T cell differentiation in lung cancer (163). Bortezomib distinctly upregulates miR-155 in CD8^+^ T cells, blocking PD-1-mediated T cell exhaustion by inhibiting the SOCS1/SHIP1 pathway, thereby improving TME in breast cancer (164). Additionally, Sevoflurane upregulates miR-124-3p to inhibit the PD-L1/Ras/ERK pathway, reducing esophageal cancer metastasis and enhancing CD8^+^ T cell cytotoxicity (60); Thalidomide inhibits FGD5-AS1 to upregulate miR-454-3p, degrade ZEB1, and downregulate PD-L1/VEGFA, effectively blocking immune escape in non-small cell lung cancer (17); N-acetylcysteine (NAC) neutralizes ROS and upregulates tumor exosome miR-155-5p to inhibit PD-L1, reduce macrophage infiltration in ovarian cancer, and activate CD8^+^ T cell (16); Astragalus polysaccharides (APS) upregulate miR-133a-3p to degrade MSN, disrupting PD-L1 stability and reversing IFNγ-induced immune suppression in hepatocellular carcinoma (165); IL-2-stimulated exosomes carrying miR-181a-3p/miR-223-3p directly downregulate PD-L1 in melanoma cells while activating CD8^+^ T cells and inhibiting Treg function (166).

TME reprogramming strategies demonstrate the potential to indirectly enhance CD8^+^ T cell activity by regulating myeloid cell and macrophage function via miRNA. NT157 inhibits TGFβRII in Gr-1^+^CD11b^+^ myeloid cells by upregulating miR-130a/145, promoting secretion of IFNγ by CD8^+^ T cells, and effectively inhibiting breast cancer metastasis (101). Poly(I:C) inhibits the conversion of Ly6C^+^ monocytes into TAMs in lung cancer by upregulating miR-155 and downregulating M-CSF, thereby reshaping the immunosuppressive microenvironment (167). OTS167 upregulates miR-505-3p to inhibit MELK, blocking the STAT3/CCL2 axis to promote M1 macrophage polarization, significantly enhancing the recruitment of CD8^+^ T cells in liver cancer (81). Radiotherapy-induced circPIK3R3 exosomes activate the macrophage IRF7/I-IFN pathway by binding to miR-872-3p, enhancing CD8^+^ T cell secretion of IFNγ/GZMB via the JAK/STAT signaling pathway, and producing a distal effect that inhibits melanoma metastasis (168).

In hormone/receptor-targeted therapy, Fulvestrant antagonizes estrogen signaling to upregulate miR-765, inhibiting the PLP2/Notch pathway, thereby not only blocking the EMT process in endometrial cancer but also enhancing CD8^+^ T cell activity (12). In strategies directly promoting T cell proliferation, Rapamycin inhibits mTOR to upregulate miR-625-3p, effectively promoting CD8^+^ T cell proliferation and reconstitution after allogeneic transplantation in AL (111).

It is worth noting that certain environmental toxins can impair CD8^+^ T cell function by disrupting the miRNA network. DCMU/Diuron induces disruption of miR-3135b/miR-21-5p in CD8^+^ T cells, inhibiting cytokine and GZMB secretion, and severely weakening antitumor cytotoxicity, highlighting the destructive effect of environmental toxins on immune surveillance (169). These studies collectively demonstrate the importance of miRNAs as key regulatory hubs for drug-mediated modulation of CD8^+^ T cell function, providing a robust theoretical foundation and clinical translation potential for the development of miRNA-based immunomodulatory strategies. **Table 8** lists 16 drugs’ miRNA, action location, regulatory pathway, etc., and their impacts on CD8+T cells and tumor therapy. Many drugs can affect CD8+T cell function by regulating miRNA, providing new strategies for tumor immunotherapy.

**Table 8:** Key information of drugs regulating miRNAs to impact tumors and CD8^+^T cell.

Drug	MicroRNA	Location	Pathway	Cancer	CD8+T cell	Result	Ref.
sitagliptin	miR-30-5p	cancer cell	DPP4↓/CXCL10	HCC	↑	efficacy of anti-PD1 therapy↑	(79)
Anti-IL-17A	miR-15b-5p	cancer cell	IL-17A↓/P65/NRF1↓/miR-15b-5p↑/PD-L1↓	COADREAD	↑	anti-PD-1 sensitivity↑ and MDSCs↓	(44)
metformin	miR-107	CD8+ T cell	AMPK↑/miR-107↓/Eomes↑/PD-1↓	LUNG	↑	cytotoxicity in CAR-T cells↑	(162)
bortezomib	miR-155	CD8+ T cell	miR-155↑/SOCS1↓/SHIP1↓/PD-1↓	BRCA	↑	T cell exhaustion in the tumor microenvironment↓	(163)
sevoflurane	miR-124-3p	cancer cell	miR-124-3p↑/Ras↓/ERK↓/PD-L1↓	ESCA	↑	xenograft growth and lung metastases *in vivo*↓	(56)
thalidomide	miR-454-3p	cancer cell	FGD5-AS1↓/miR-454-3p↑/ZEB1↓/PD-L1↓	NSCLC	↑	NSCLC angiogenesis and immune evasion↓	(17)
NAC	miR-155-5p	tumor exosome	ROS↓/miR-155-5p↑/PD-L1↓	OV	↑	macrophage migration/infiltration↓	(16)
APS	miR-133a-3p	cancer cell	miR-133a-3p↑/MSN↓/IFN-γ↓/PD-L1↓	HCC	↑	reversed IFN-γ-induced PD-L1 upregulation	(164)
IL2-sEVs	miR-181a-3p/miR-223-3p	cancer cell/CD8+ T cell	miR-181a-3p, miR-223-3p↑/PD-L1↓	SKCM	↑	melanoma sensitivity to CD8^+^ T cells↑and Treg activity↓	(165)
NT157	miR-130a/145	Gr-1^+^CD11b^+^	IGF1R↓	BRCA	↑	anti-tumor immunity↑and metastasis↓	(101)
poly(I:C)	miR-155	Ly6C+ monocyte	miR-155↑/M-CSF↓ STAT3,M-CSF↑/Arg1↑	LUNG	↑	monocytes transition into TAMs↓	(166)
OTS167	miR-505-3p	cancer cell	miR-505-3p↑/MELK↓/pSTAT3,CCL2↓	HCC	↑	M1 macrophage polarization↑and M2 polarization↓	(62)
RT	miR-872-3p	tumor-derived exosome	circPIK3R3/miR-872-3p/IRF7I-IFN↑(macrophagel )→ JAK/STAT↑(CD8+ T cell)/IFN-γ, GZMB↑	SKCM	↑	primary tumors and lung metastases↓	(167)
fulvestrant	miR-765	cancer cell	Erβ↓/miR-765↑/PLP2↓/Notch↓	UCEC	↑	alleviates estrogen-mediated miR-765 downregulation block EMT and proliferation	(12)
rapamycin	miR-625-3p	CD8+T cells	mTOR↓/miR-625-3p↑	AL	↑	proliferation level of CD8+ T cells after Allo-HSCT↑	(115)
DCMU/Diuron	hsa-miR-3135b/hsa-miR-21-5p	CD8+ T cell	miRNA dysregulation/cytokine and granzyme B↓	–	↓	CD8+ T cells’ cytotoxic activity directed against cancer cells↓	(168)

This table summarizes key information on pharmacological agents that modulate microRNA (miRNA) expression to influence tumor behavior and CD8^+^ T cell activity. It details how various drugs alter specific miRNA levels in different cellular locations, subsequently regulating critical pathways—particularly the PD-1/PD-L1 axis—across multiple cancer types. These interventions primarily enhance CD8^+^ T cell function and reverse immune suppression, thereby improving responses to anti-PD-1 therapy and other immunotherapies.

Sitagliptin, sitagliptin; Anti-IL-17A, anti-interleukin-17A antibody; metformin, metformin; bortezomib, bortezomib; sevoflurane, sevoflurane; thalidomide, thalidomide; NAC, N-acetylcysteine; APS, astragalus polysaccharide; IL2-sEVs, interleukin-2-engineered small extracellular vesicles; NT157, NT157; poly(I:C), polyinosinic-polycytidylic acid; OTS167, OTS167; RT, radiotherapy; fulvestrant, fulvestrant; rapamycin, rapamycin; DCMU/Diuron, 3-(3,4-dichlorophenyl)-1,1-dimethylurea/diuron; HCC, hepatocellular carcinoma; COADREAD, colorectal adenocarcinoma; LUNG, lung cancer; BRCA, breast cancer; ESCA, esophageal carcinoma; NSCLC, non-small cell lung cancer; OV, ovarian cancer; SKCM, skin cutaneous melanoma; UCEC, uterine corpus endometrial carcinoma; AL, acute leukemia. CD8^+^ T cell column: ↑, change that confers a benefit to CD8^+^ T cells (e.g., enhanced function, survival, or proliferation); ↓, change that is harmful to CD8^+^ T cells (e.g., impaired function or reduced viability).

### 3.3 Nucleic acid drug

Nucleic acid drugs have opened up new avenues for tumor immunotherapy by precisely regulating the miRNA network, with antisense oligonucleotides (ASOs) and siRNA technology showing great potential in reshaping T cell function. **Table 9** lists key info of 5 non-miRNA RNAs; nucleic acid drugs regulate miRNA networks for tumor immunotherapy. In directly regulating immune-suppressive cells, miR-126-specific ASOs downregulate miR-126 expression, upregulate p85β, and activate the PI3K-Akt pathway, thereby inhibiting the expression of Foxp3. This significantly reduces the induction and immune-suppressive function of Tregs in breast cancer while alleviating the suppression of CD8^+^ T cells, effectively enhancing the anti-tumor immune response (106). Conversely, abnormal expression of certain non-coding RNAs can lead to resistance to immunotherapy. For example, upregulation of tRF-3024b in esophageal squamous cell carcinoma inhibits miR-192-5p and upregulates BCL-2, not only promoting tumor cell anti-apoptotic capacity but also increasing resistance to ICIs, while also inhibiting CD8^+^ T cell activity, revealing the negative regulatory mechanism of the drug-resistant TME on T cells (58).

**Table 9:** Key information of non-miRNA RNAs regulating miRNAs and affecting tumors & CD8^+^T cells.

RNA (except microRNA)	MicroRNA	Location	Pathway	Cancer	CD8+T cell	Result	Ref.
miR-126 ASO	miR-126	Tregs	ASO/miR-126↓/p85β↑/PI3K-Akt↑/Foxp3↓	BRCA	↑	induction and suppressive function of Tregs↓	(106)
tRF-3024b	miR-192-5p	cancer cell	tRF-3024b↑/miR-192-5p↓/BCL-2↑	ESCC	↓	drug resistance against ICIs↑	(57)
IDO small interfering RNA	miR-143	Tcm CD8+ cell	IDO↓/miR-143↑/Glut-1↑	ESCA	↑	memory T cell differentiation and metabolism reprogramming through Glut-1↑	(64)
mimics, inhibitors, overexpression vectors or siRNAs	miR-195-5p	cancer cell	LINC00473↓/miR-195-5p↑/PD-L1↓	PAAD	↑	apoptosis, proliferation, invasion, and migration of the cancer cells↑	(54)
FOXD3-AS1 siRNA	miR-2682	exosome	FOXD3-AS1↓/EIF3B↑/miR-2682↑/RBM39↓	LUNG	↑	cancer cell growth and inhibition of apoptosis↓	(18)

This table summarizes how non-miRNA RNAs (including ASOs, siRNAs, and tRNA fragments) regulate specific miRNAs to influence tumors and CD8^+^ T cells. These regulatory RNAs modulate key pathways such as PD-L1 expression and T cell metabolism, primarily by targeting and suppressing immunosuppressive miRNAs. The resulting effects enhance CD8^+^ T cell function, inhibit tumor growth, and overcome therapy resistance, demonstrating their potential as therapeutic tools in cancer immunotherapy.

miR-126 ASO, microRNA-126 antisense oligonucleotide; tRF-3024b, tRNA-derived fragment 3024b; IDO small interfering RNA, indoleamine 2,3-dioxygenase small interfering RNA; mimics, inhibitors, overexpression vectors or siRNAs, microRNA-195-5p regulatory tools; FOXD3-AS1 siRNA, FOXD3 antisense RNA 1 small interfering RNA; BRCA, breast cancer; ESCC, esophageal squamous cell carcinoma; ESCA, esophageal carcinoma; PAAD, pancreatic adenocarcinoma; LUNG, lung cancer. CD8^+^ T cell column: ↑, change that confers a benefit to CD8^+^ T cells (e.g., enhanced function, survival, or proliferation); ↓, change that is harmful to CD8^+^ T cells (e.g., impaired function or reduced viability).

In T cell metabolic reprogramming strategies, the siRNA delivered by targeting indoleamine 2, 3-dioxygenase (IDO), which inhibits IDO expression and upregulates miR-143, thereby increasing the expression of the glucose transporter Glut-1, successfully drives the metabolic reprogramming of Tcm, enhances glycolytic activity, promotes memory T cell differentiation, and establishes long-term anti-tumor immunity (59). Indirect regulatory strategies targeting PD-L1 have also made significant progress. By downregulating the lncRNA lnc00473 in pancreatic cancer using miRNA mimics or siRNA, miR-195-5p is upregulated, and PD-L1 expression is inhibited. This not only directly suppresses tumor cell proliferation and invasion but also alleviates the inhibitory effect of PD-L1 on CD8^+^ T cells, which significantly enhances T cell infiltration (84).

**Figure 6:**
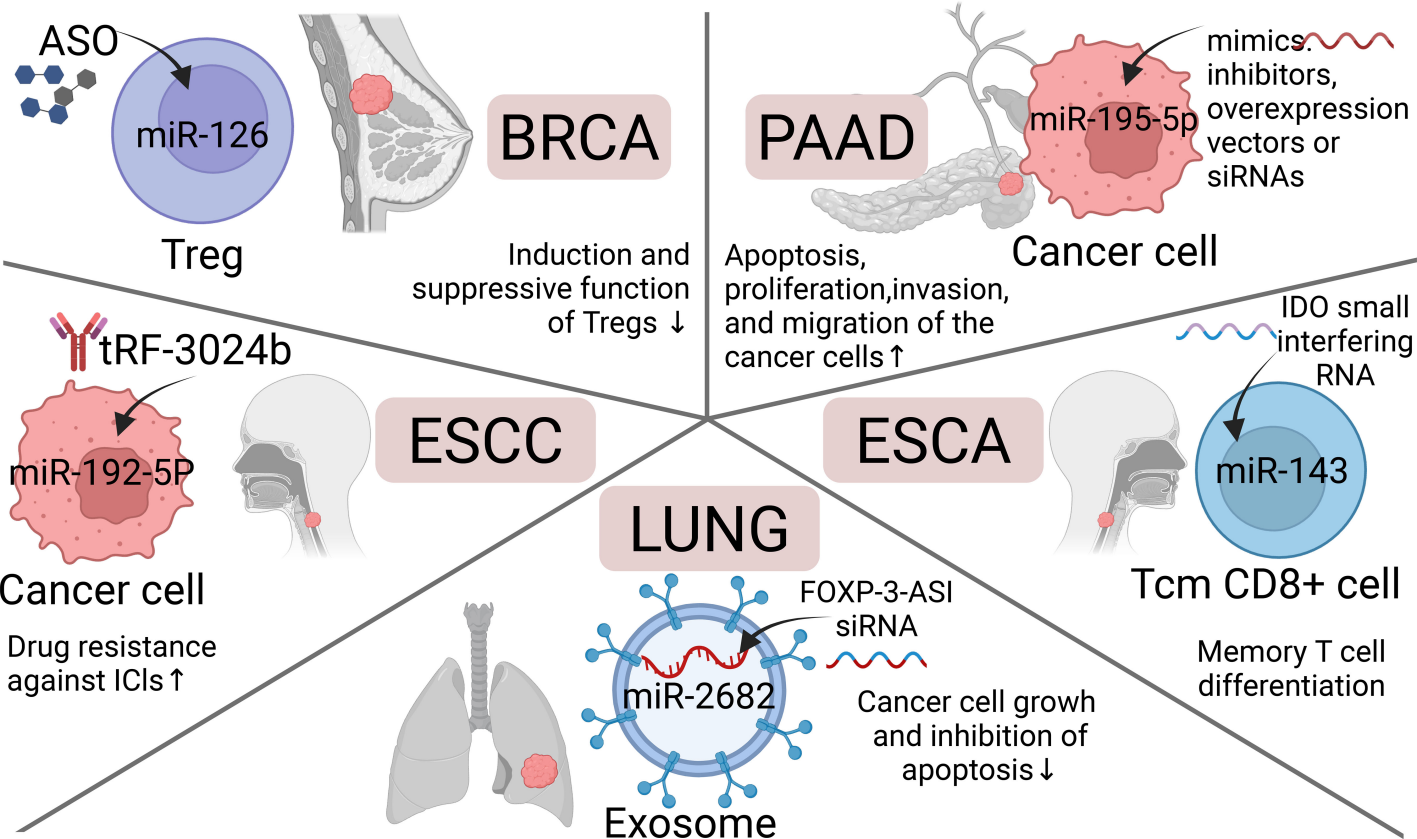
Nucleic acid drug-mediated miRNA network regulation in tumor immunotherapy across multiple cancer types. In BRCA, ASO targets miR-126 in Tregs to reduce their suppressive function. In PAAD, mimics/inhibitors modulate miR-195-5p in cancer cells, affecting apoptosis and proliferation. In ESCA, IDO siRNA regulates miR-143 in Tcm CD8+ cells to promote memory T cell differentiation. In LUNG, exosomal miR-2682 with FOXP-3-AS1 siRNA inhibits cancer cell growth. In ESCC, tRF-3024b modulates miR-192-5p in cancer cells, increasing ICI resistance. These strategies demonstrate nucleic acid drugs’ potential in reshaping tumor immunity via miRNA regulation.

The application of exosome delivery systems further expands the therapeutic potential of nucleic acid therapeutics. FOXD3-AS1 siRNA delivered to lung cancer cells through exosomes downregulates FOXD3-AS1, thereby upregulating EIF3B. This cascade regulation subsequently promotes miR-2682 expression and inhibits RBM39. This mechanism not only directly suppresses lung cancer cell growth and induces apoptosis but also indirectly enhances the antitumor activity of CD8^+^ T cells (18). These studies systematically demonstrate diverse strategies by which nucleic acid therapeutics remodel the tumor immune microenvironment through regulating miRNA-lncRNA networks, which provide crucial theoretical foundations and technical support for developing nucleic acid-based precision immunotherapies. **Figure 6** illustrates nucleic acid drugs regulating miRNA networks in various tumors for immunotherapy.

## 4 Conclusion

This review synthesizes current research on miRNA–CD8+ T cell interactions in tumor immunity. It systematically summarizes key regulatory pathways and therapeutic potential across cancer types. By integrating data from multiple studies, we explore how miRNAs regulate CD8+ T cell activation, function, and TME interactions. We also identify research gaps and future directions. To provide a structured overview, we present three complementary tables. **Table 10** highlights pan-cancer miRNAs with conserved roles in regulating CD8+ T cell function across multiple tumor types. **Table 11** lists high-confidence miRNA targets whose mechanistic roles have been validated by independent studies. A detailed classification of miRNAs by cellular origin—whether derived from CD8+ T cells (direct regulators) or other TME cell types (indirect regulators)—is provided in the accompanying comprehensive tables.

**Table 10:** Pan-cancer miRNAs: key regulators of CD8+ T cell function with conserved roles across multiple cancer types.

miRNA	Core regulatory function	Key pathway	Cancer types	Therapeutic implication
miR-155	Promotes T-cell function; drives exhaustion if dysregulated	SOCS1/SHIP1→STAT5/Akt; PD-L1	SKCM, BRCA, DLBC, NSCLC, LIHC	Enhance in T cells; inhibit in tumor cells
miR-34a	Tumor suppressor; loss upregulates PD-L1, exhausts T cells	PD-L1 regulation; T-cell proliferation	LUAD, STAD, SKCM, BRCA, LIHC	Restore to boost anti-PD-1/PD-L1 therapy
miR-200 family	Regulates EMT & immune evasion via ZEB1/PD-L1	ZEB1/miR-200 loop→PD-L1	NSCLC, COADREAD, OV, LIHC	Target axis to block metastasis & immune escape
miR-21	Oncogenic; drives immunosuppressive microenvironment	PD-L1; M2 polarization; Breg induction	NSCLC, BRCA, HNSCC, ESCA, COADREAD	Inhibit to relieve immunosuppression
miR-15/16	Regulate T-cell exhaustion & immune checkpoints	mTOR; anti-apoptotic genes	GBM, PRAD, CML	Modulate to reverse exhaustion
miR-148a	Dual regulator of MHC-I antigen presentation & PD-L1	ZEB1/Rab6A→PD-L1; CANX/MHC-I	LUAD, COADREAD, THCA	Restore to enhance visibility & reduce suppression
miR-206	Promotes M1 polarization & CD8+ recruitment; inhibits Tregs	KLF4/CCL2/CCR2; KRAS-MEK-ERK/TGFβ1	LIHC, HNSCC	Remodel innate & adaptive immunity
miR-326	Reverses immunosuppression via TGF-β1 targeting	SMO-Gli2/TGF-β1; circ001678/ZEB1/PD-L1	NSCLC, GBM, DLBC	Target to enhance CAR-T efficacy

This table highlights miRNAs that are repeatedly implicated in similar regulatory pathways (e.g., PD-1/PD-L1 axis, T-cell exhaustion) across diverse tumor types, suggesting they are core, fundamental regulators of the tumor immune microenvironment.

**Table 11:** High-confidence miRNA targets: independently validated by multiple studies.

miRNA	Reported role in CD8+ T-cell immunity	Validating references	Level of evidence
miR-155	Promotes function via cytokine response & SOCS1/SHIP1 inhibition	Dudda 2013 (141); Ji 2015 (142); Martinez-Usatorre 2019 (143); Ji 2019 (145); Monnot 2020 (144)	`
miR-34a	Loss upregulates PD-L1, driving T-cell exhaustion	Li 2022 (13); Wang 2020 (14); Ping 2018 (65); Wang 2023 (139); Hart 2020 (61)	High
miR-200c/200	Suppresses PD-L1 via ZEB1; loss promotes immune escape	Chen 2014 (15); Xu 2023 (77); Sun 2018 (52)	High
miR-21	Drives immunosuppressive TME via PD-L1, Bregs, M2 macrophages	Yin 2022 (43); Guo 2022 (89); Miao 2015 (34); Samiei 2022 (78); He 2017 (70)	High
miR-142-5p/142	Induces T-cell exhaustion & immune privilege; mutations drive leukemogenesis (CESC, AML)	Zhou 2021 (102); Kawano 2023 (121)	Moderate-High
miR-326	Enhances immunity by inhibiting TGF-β1 pathway (GBM, NSCLC, DLBC)	Li 2017 (125); Tian 2022 (16); Xu 2020 (10)	Moderate
miR-181a	Regulates T-cell survival/apoptosis; involved in STAT3 networks (leukemia, melanoma)	Assmann 2022 (117); Jung 2022 (165)	Moderate
miR-148a	Silences MHC-I pathway, impairing CD8+ T-cell attack (COADREAD, THCA, LUAD)	Zheng 2021 (86); Wang 2021 (85); Xiao 2023 (36)	Moderate-High

This table lists miRNAs for which the mechanistic role in CD8+ T-cell immunity has been corroborated by at least two independent research groups, increasing the robustness of the finding and its potential for clinical translation.

At the mechanistic level, miRNAs regulate CD8+ T cells through two primary modes of action. First, CD8+ T cell-derived miRNAs directly regulate intrinsic cellular functions. These include proliferation (e.g., miR-17–92 cluster promoting Type 1 T cell polarization in GBM), effector function (e.g., miR-155–IFN-γ axis enhancing antitumor responses in SKCM), exhaustion (e.g., miR-15b/16–PD-1 pathway modulating checkpoint molecules), and memory formation (e.g., let-7 family promoting memory CD8+ T cell differentiation in SKCM). As detailed in the comprehensive tables, miRNAs such as miR-491, miR-625-3p, and miR-23a show context-dependent expression changes in CD8+ T cells across COADREAD, AL, and LUNG cancers. These changes directly impact cytokine production, cytotoxic activity, and T-cell senescence.

Second, non-CD8+ T cell-derived miRNAs indirectly modulate CD8+ T cell activity through TME-mediated regulation. Cancer cell-derived miRNAs (e.g., miR-27a, miR-148a-3p, miR-200c) upregulate PD-L1 or downregulate MHC-I, impairing tumor immunogenicity and CD8+ T cell infiltration in COADREAD, BRCA, and LIHC. Vesicle-derived miRNAs (e.g., exosomal miR-21-5p, miR-1246) promote immunosuppressive macrophage polarization and T-cell dysfunction in COADREAD and STAD. Other immune cell-derived miRNAs, such as miR-125a-3p from TAMs and miR-125b-5p from Tregs, further suppress CD8+ T cell function in COAD. Notably, PD-L1/PD-1 axis regulation is the most frequently reported mechanism. It involves core regulators like miR-34a, miR-200 family, and miR-148a (highlighted in **Table 10**). However, other pathways exhibit tumor-type specificity. For example, miR-206 promotes M1 macrophage polarization and CD8+ T cell recruitment in LIHC and HNSCC. In contrast, miR-326 reverses TGF-β-mediated immunosuppression in GBM, NSCLC, and DLBC (**Table 11**).

The high-confidence miRNAs listed in **Table 11**—including miR-155, miR-34a, miR-200c, miR-21, miR-142-5p, miR-326, miR-181a, and miR-148a—represent particularly robust targets. Their mechanistic roles have been corroborated by at least two independent research groups. This independent validation increases their translational potential. For instance, miR-148a silences the MHC-I pathway and impairs CD8+ T cell-mediated attack across COADREAD, THCA, and LUAD. This suggests a generalizable immune evasion strategy. Similarly, miR-155 plays a dual role: it promotes T-cell function but drives exhaustion when dysregulated. This finding is well-established across SKCM, BRCA, and DLBC, making miR-155 a key immunomodulatory target.

In therapeutic applications, miRNA-based strategies show great potential. As outlined in **Table 1**, restoring tumor-suppressive miRNAs like miR-34a could simultaneously inhibit tumor growth and enhance anti-PD-1/PD-L1 immunotherapy. Inhibiting oncogenic miRNAs like miR-21 could relieve immunosuppression and boost other therapies. Small-molecule modulators are emerging: metformin upregulates miR-34a, while sitagliptin inhibits miR-155. Nucleic acid drugs, including antisense ASOs targeting miR-21 and miR-200c-siRNA conjugates, are under investigation. Combination therapies, such as miRNA inhibitors with PD-1 antibodies, have shown synergistic effects in mouse models. Notably, miR-326 counteracts TGF-β-mediated suppression, making it a valuable target for enhancing CAR-T cell therapies. MiR-206 also represents a promising candidate for combination therapy by remodeling both innate and adaptive immune compartments.

However, clinical translation remains challenging. First, over 70% of studies focus on melanoma, lung cancer, or colorectal cancer. Data on rare tumors like sarcomas or gliomas are limited. Second, delivery systems require further optimization for targeting, stability, and safety. Third, multiple miRNAs (e.g., miR-155, miR-34a, miR-200 family) regulate overlapping targets (e.g., PD-L1, SOCS1, ZEB1). This raises questions about the feasibility of single-miRNA intervention. The complexity and redundancy of miRNA networks may limit single-target efficacy. As shown in **Table 1**, multiple miRNAs converge on the PD-1/PD-L1 axis. Off-target effects and long-term safety also remain urgent issues. Furthermore, tumor heterogeneity across patients necessitates personalized treatment strategies.

To overcome these limitations, we recommend prioritizing the following initiatives. First, expand mechanistic research to understudied tumors (e.g., pancreatic cancer, sarcomas) and CD8+ T cell subsets (e.g., tissue-resident memory cells), guided by the pan-cancer regulators in **Table 10**. Second, develop advanced delivery systems for precise spatiotemporal control of miRNA expression. Third, explore miRNA combination therapies to overcome tumor heterogeneity through multi-target intervention—potentially targeting multiple high-confidence miRNAs from **Table 11** simultaneously. Fourth, establish a biomarker system based on miRNA expression profiles to guide personalized treatment decisions. Fifth, accelerate clinical translation through rigorous trials validating safety and efficacy. As our understanding of miRNA function deepens and technological platforms mature, miRNA-based immunotherapy—informed by the conserved regulators and validated targets summarized here—holds promise as a powerful tool in the fight against cancer.

## Glossary

AL: acute leukemia
Allo-HSCT: allogeneic hematopoietic stem cell transplantation
AML: acute myeloid leukemia
APS: astragalus polysaccharide
ASO: antisense oligonucleotide
BLCA: bladder urothelial carcinoma
BRCA: breast cancer
CAR-T: chimeric antigen receptor T-cell
CML: chronic myeloid leukemia
COAD: colon adenocarcinoma
COADREAD: colorectal adenocarcinoma
CR: complete remission
CTCL: cutaneous T-cell lymphoma
DC: dendritic cell
DCMU: 3-(3, 4-dichlorophenyl)-1, 1-dimethylurea
DLBC: diffuse large B-cell lymphoma
EEA: endometrioid endometrial adenocarcinoma
EMT: epithelial-mesenchymal transition
ESCA: esophageal carcinoma
ESCC: esophageal squamous cell carcinoma
GBM: glioblastoma
HCC: hepatocellular carcinoma
HDI: hydrodynamic injection
HGSC: high-grade serous ovarian carcinoma
HNSCC: head and neck squamous cell carcinoma
ICC: intrahepatic cholangiocarcinoma
ICIs: immune checkpoint inhibitors
IDO: indoleamine 2, 3-dioxygenase
IONR: iron oxide nanorods
KIRC: kidney renal clear cell carcinoma
LAML: acute myeloid leukemia
LC: laryngeal cancer
LDHs: layered double hydroxides
LIHC: liver hepatocellular carcinoma
LNP: lipid nanoparticles
LSCC: lung squamous cell carcinoma
LUAD: lung adenocarcinoma
LUNG: lung cancer
NAC: N-acetylcysteine
NPC: nasopharyngeal carcinoma
NSCLC: non-small cell lung cancer
ORR: objective response rate
OS: osteosarcoma
OSCC: oral squamous cell carcinoma
OV: ovarian cancer
PAAD: pancreatic adenocarcinoma
PDAC: pancreatic ductal adenocarcinoma
PRAD: prostate adenocarcinoma
PTC: papillary thyroid carcinoma
R/R B-ALL: relapsed/refractory B-cell acute lymphoblastic leukemia
SKCM: melanoma
STAD: stomach adenocarcinoma
TAM: tumor-associated macrophage
TEX: tumor-derived exosomes
THCA: thyroid carcinoma
TILs: tumor-infiltrating lymphocytes
T-LGLL: T-cell large granular lymphocytic leukemia
TME: tumor microenvironment
TNBC: triple-negative breast cancer
UCEC: uterine corpus endometrial carcinoma.
